# Future Exploration of the Outer Heliosphere and Very Local Interstellar Medium by Interstellar Probe

**DOI:** 10.1007/s11214-022-00943-x

**Published:** 2023-02-28

**Authors:** P. C. Brandt, E. Provornikova, S. D. Bale, A. Cocoros, R. DeMajistre, K. Dialynas, H. A. Elliott, S. Eriksson, B. Fields, A. Galli, M. E. Hill, M. Horanyi, T. Horbury, S. Hunziker, P. Kollmann, J. Kinnison, G. Fountain, S. M. Krimigis, W. S. Kurth, J. Linsky, C. M. Lisse, K. E. Mandt, W. Magnes, R. L. McNutt, J. Miller, E. Moebius, P. Mostafavi, M. Opher, L. Paxton, F. Plaschke, A. R. Poppe, E. C. Roelof, K. Runyon, S. Redfield, N. Schwadron, V. Sterken, P. Swaczyna, J. Szalay, D. Turner, H. Vannier, R. Wimmer-Schweingruber, P. Wurz, E. J. Zirnstein

**Affiliations:** 1 The Johns Hopkins University Applied Physics Laboratory, Laurel, MD USA; 2 University of California Berkeley, Berkeley, CA USA; 3 Office of Space Research and Technology, Academy of Athens, Athens, 10679 Greece; 4 Southwest Research Institute, San Antonio, TX USA; 5 Laboratory for Atmospheric and Space Physics, University of Colorado at Boulder, Boulder, CO USA; 6 University of Illinois Urbana-Champaign, Urbana, IL USA; 7 University of Bern, Bern, Switzerland; 8 Imperial College London, London, UK; 9 ETH, Zurich, Switzerland; 10 University of Iowa, Iowa City, IA USA; 11 University of Colorado Boulder, Boulder, CO USA; 12 Space Research Institute, Austrian Academy of Sciences, Graz, Austria; 13 University of New Hampshire, Durham, NH USA; 14 Boston University, Boston, MA USA; 15 Technical University Braunschweig, Braunschweig, Germany; 16 Weslayan University, Middeltown, CT USA; 17 Princeton University, Princeton, NJ USA; 18 University of Kiel, Kiel, Germany; 19 Planetary Science Institute, Tucson, AZ USA

**Keywords:** Outer heliosphere, Interstellar medium, Interstellar probe

## Abstract

A detailed overview of the knowledge gaps in our understanding of the heliospheric interaction with the largely unexplored Very Local Interstellar Medium (VLISM) are provided along with predictions of with the scientific discoveries that await. The new measurements required to make progress in this expanding frontier of space physics are discussed and include in-situ plasma and pick-up ion measurements throughout the heliosheath, direct sampling of the VLISM properties such as elemental and isotopic composition, densities, flows, and temperatures of neutral gas, dust and plasma, and remote energetic neutral atom (ENA) and Lyman-alpha (LYA) imaging from vantage points that can uniquely discern the heliospheric shape and bring new information on the interaction with interstellar hydrogen. The implementation of a pragmatic Interstellar Probe mission with a nominal design life to reach 375 Astronomical Units (au) with likely operation out to 550 au are reported as a result of a 4-year NASA funded mission study.

## Introduction

Our Star and its protective heliosphere are one of a hundred billion stars and astrospheres in the galaxy that plow through the vast interstellar medium made up of the material from supernova remnants and condensed stellar blow off. During its evolution, the Sun has completed about twenty revolutions around the galactic core and has encountered widely different environments that have all contributed to the evolution of the system we live in (Linsky et al. 2022). Large differences in interstellar plasma and gas densities, charge fractions, temperatures and magnetic fields have dramatically impacted the heliospheric interaction and size from many times larger than today to a severely compressed heliosphere likely well below the orbit of the inner planets. These long-term dynamics have had dramatic consequences for the penetration of interstellar material, dust and galactic cosmic rays (GCRs) that we are all made of, and have affected elemental and isotopic abundances, atmospheric evolution and perhaps even conditions for habitability (Opher and Loeb 2022). Along this 4.6-Gyear evolutionary journey several supernovae have occurred within 100 pc of the Sun, with the most recent one occurring only 3 million years ago that resulted in a full exposure of the inner solar system to the unshielded interstellar environment and GCRs.

The Local Interstellar Medium (LISM) is defined as the region of space containing material to the nearest $10^{19}$ H atoms per $\text{cm}^{2}$ (Cox and Reynolds 1987) and ranges 30–200 pc from the Sun. The VLISM, on the other hand, is defined here as the region within 0.01 pc, or 2063 au, of the Sun (Holzer 1989) and therefore includes the environment unperturbed by the helisophere. Other definitions exist, such as the one by Zank (2015), who defined it as region “surrounding the Sun that is modified by the deposition of heliospheric material”.

**Fig. 1:**
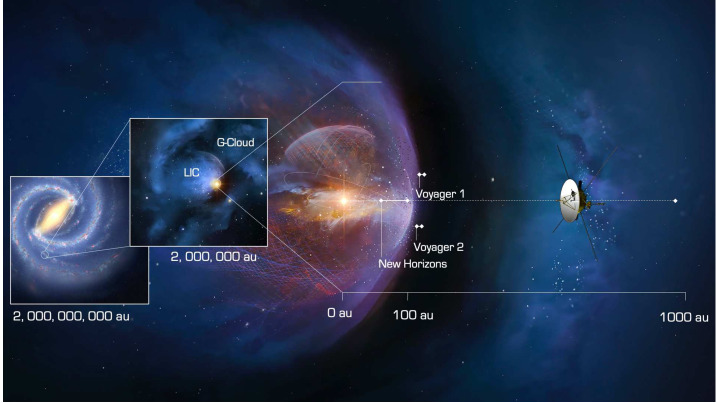
The outer heliosphere and VLISM are almost completely unexplored regions of space, where a new regime of physical processes is responsible for upholding our habitable astrosphere. Interstellar Probe on a fast escape trajectory would bring new understanding of how our star interacts with its surrounding VLISM to ultimately understand the evolutionary path of the solar system through the dramatically different environments in the galaxy

The very little that is known about the LISM reveals a remarkable coincidence. Only 60,000 years ago, the Sun entered the Local Interstellar Cloud (LIC). For the past several thousand years the Sun appears to have traversed the outer layer of the LIC, and today is located at the very edge of it (Fig. 1). Within mere thousands of years the heliosphere will find itself in the neighboring G-cloud with a very different interstellar environment that could, again, dramatically alter our heliosphere.

Complex interactions of plasma, magnetic field, and neutral interstellar gas starting near the Sun and acting throughout the heliosphere are responsible for the entire boundary interaction with the VLISM. Of the five spacecraft with solar-system escape speeds, only Voyager 1 and 2 have taken measurements through the termination shock (TS), the heliosheath and the heliopause (HP) (e.g. Decker et al. 2005, 2008; Krimigis et al. 2011; Krimigis et al. 2013; Krimigis et al. 2019; Dialynas et al. 2022) and are expected to operate until 2030. Although designed as planetary flyby missions, their limited payload uncovered a range of mysteries that have made it clear that the heliospheric boundary represents a new regime of space physics. New Horizons is now the only operating spacecraft in the outer heliosphere and is expected to operate through the TS and well into the heliosheath. Therefore, its measurements of plasma, energetic particles, Ly-alpha and dust are becoming increasingly important for heliophysics.

Given the far-reaching implications for understanding our home in the galaxy and the very limited information at hand, the outer heliosphere and the VLISM represent perhaps one of the most rewarding and least explored frontiers of space physics. Science observations from a future spacecraft on a fast escape trajectory through the heliosphere into the VLISM would offer a snapshot in time of the heliosphere along its evolutionary journey around the galaxy necessary to understand the current state of its global interaction and nature, to understand ultimately where our home came from and where we are going.

**Fig. 2:**
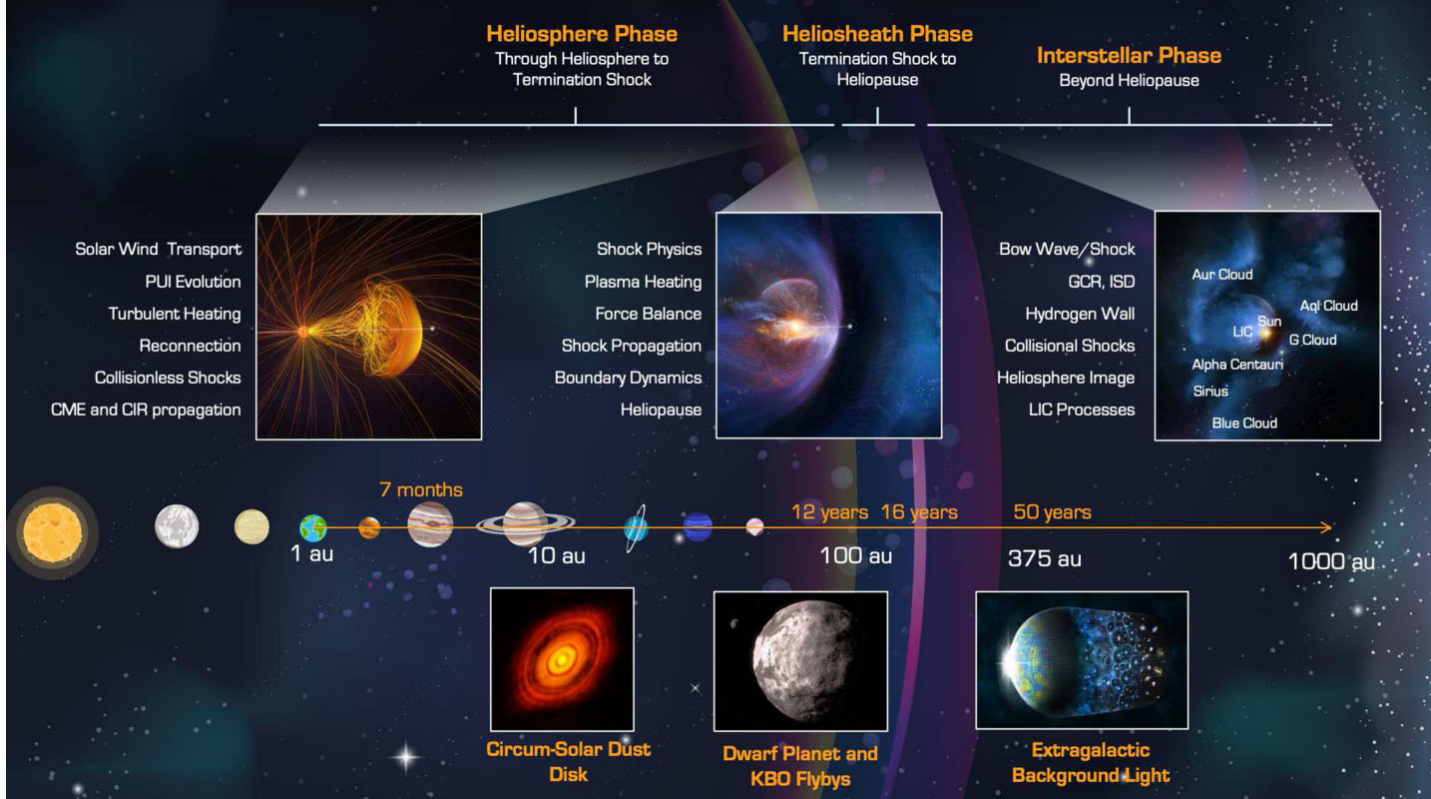
Understanding our habitable astrosphere and its home in the galaxy requires science observations starting near the Sun and out through the heliospheric boundary and ultimately out into the unexplored VLISM. Such an investigation would not only span several of the sub-disciplines of solar and space physics, but would also offer natural opportunities for astrophysics and planetary sciences

The sections in this article provide an overview of the current knowledge of the heliosphere and the VLISM, the outstanding science questions, and the needed science measurements that start in the inner heliosphere, throughout the heliosphere, out through the heliosheath and into the unexplored VLISM (Fig. 2). The article concludes with a brief description of the results of a four-year, NASA-funded study on the pragmatic implementation of a future Interstellar Probe (McNutt et al. 2022; Brandt et al. 2022) with launch windows opening in 2036 to propel a spacecraft with a dedicated payload three times farther than what Voyager 1 will explore (Mission Concept Report available here). As such, a future Interstellar Probe would mark the first explicit step into the galaxy with robotic exploration and the beginning of a new realm of space physics.

## The Heliosphere in the VLISM: A New Regime of Space Physics

### The Solar Wind and the Critical Role of PUIs

**Fig. 3:**
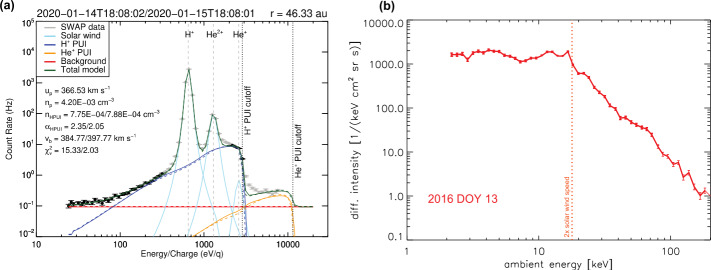
(**a**) Proton PUI measurements by SWAP halfway to the termination shock by New Horizons (McComas et al. 2021). (**b**) $\text{He}^{+}$ PUI Measurements by PEPSSI

The interaction with the VLISM already starts deep inside the inner heliosphere near the Sun. Here, the neutral interstellar gas that permeates the heliosphere is ionized by photo- and electron-impact ionization as well as charge-exchange processes creating suprathermal interstellar pickup ions (PUIs), as first observed for helium on AMPTE IRM (Möbius et al. 1985) and for hydrogen on Ulysses (Gloeckler et al. 1993). PUIs are “picked up” by the solar wind convection electric field and rapidly accelerated up to twice the solar wind speed. PUIs are also formed from interaction with an “inner source” of dust grains near the Sun and the solar wind (Geiss et al. 1995). Unfortunately, neither of the Voyager spacecraft carried instrumentation for measuring PUIs. However, New Horizons is equipped with the Solar Wind Around Pluto (SWAP) (McComas et al. 2008) and the Pluto Energetic Particle Spectrometer Science (PEPSSI) (McNutt et al. 2008) that measure the important proton and $\text{He}^{+}$ PUIs (Fig. 3).

Following the previous discovery by Voyager 2 of solar wind heating and deceleration in the outer heliosphere (Richardson and Smith 2003), New Horizons confirmed a noticeable slowdown of the solar wind at $\sim30~\text{au}$ due to the mass-loading of PUIs (Elliott et al. 2019). The temperature profile of core solar wind ions was well above what is expected for an adiabatic profile, which is consistent with turbulent heating caused by the initially unstable ring-beam distributions of newly born PUIs that indirectly heat the solar wind as they are scattered by low-frequency turbulence (Zank et al. 2018). In the outer heliosphere, it is now evident from New Horizons observations that the PUIs dominate the thermal pressure by an order of magnitude over the solar wind thermal pressure and magnetic pressure (McComas et al. 2021).

The TS transition by Voyager 1 (at 94 au from the Sun in 2004; Stone et al. 2005) and Voyager 2 (at 84 au in 2007; Stone et al. 2008) marked the first signatures of the edges of the outer heliosphere. While the TS was anticipated to be a strong shock, the observed changes in plasma showed a weak shock (Richardson et al. 2008), almost absent of heating of the solar wind plasma. It almost came as a complete surprise that the solar wind flow downstream of the TS remained supersonic with respect to thermal ions. Unlike planetary bow shocks, the TS is mediated not by thermal plasma populations but instead by the suprathermal PUIs (Mostafavi et al. 2017, 2018b). This behavior was also observed at interplanetary shocks by the New Horizons’ SWAP instrument (McComas et al. 2021; Zirnstein et al. 2018).

**Fig. 4:**
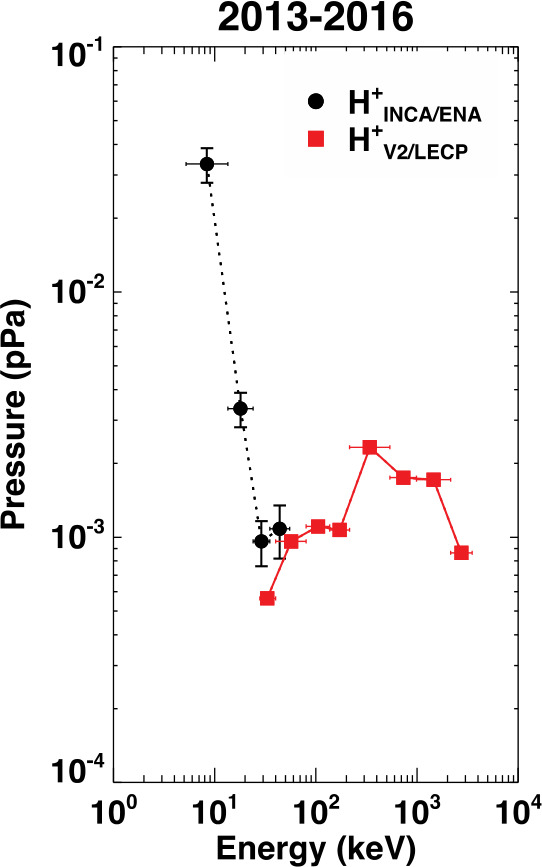
PUIs and suprathermal particles dominate the total pressure in the heliosheath as can be derived from remote ENA observations by Cassini/INCA in the 5–55 keV range compared to Low-Energy Charged Particle (LECP) measurements at higher energies along Voyager 2’s trajectory through the heliosheath (from Dialynas et al. 2019)

As the solar wind crosses from upstream (closer to the Sun) to downstream (farther from the Sun) across the TS, the magnetic field strength and temperature suddenly increase with a corresponding sudden decrease in the flow speed (Li et al. 2008) by a factor of $\sim2.5$ predicted by the Rankine–Hugoniot jump conditions. However, because of the nature of this shock, the plasma density observed by Voyager 2 increases only by a factor of $\sim2$ (Li et al. 2008). Once the PUI-loaded solar wind interacts with, and flows across the TS, the PUI population dominates the force balance in the heliosheath (Fig. 4) and at the heliopause (HP) against the apparent flow of the VLISM (Rankin et al. 2019a; Dialynas et al. 2020, 2019).

**Fig. 5:**
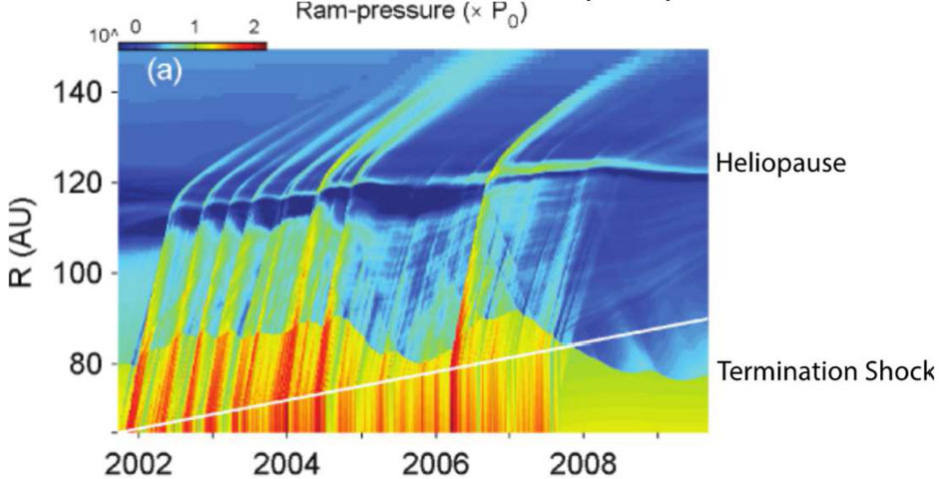
Simulation showing the responses of thermal pressure to propagating disturbances along the Voyager-2 direction (Washimi et al. 2011). The TS responds to the solar wind dynamic pressure pulses moving several astronomical units outward and inward. Solar wind shocks and waves create highly dynamic flows in the heliosheath. Note how also the HP responds to the disturbances but with much smaller amplitude. The white line denotes the Voyager 2 trajectory

Many open questions and arguments still exist due to the lack of complete measurements, including particle distributions, magnetic fields and coordinated observations of wave-particle interactions. The general problem is to determine the dissipation processes in a plasma that comprises a suprathermal PUI distribution embedded in a cold Maxwellian plasma.

### Interstellar Neutral Interactions

While interstellar electrons and ions flow around the HP, the interstellar neutral gas propagates inside the heliosphere and dramatically affects the solar wind energetics in the outer heliosphere and governs the size of the heliosphere. The neutral gas mainly consists of H atoms ($\sim90\%$) with a range of minor species (e.g., He, O, N, Ne, Ar, and other elements) (Gloeckler et al. 2009; Geiss and Gloeckler 2003). The effectiveness of the passage of elements through the heliosphere boundary and the depth to which they can advance into the heliosphere depends on atomic properties. Because of coupling of neutral atoms with plasma, atoms are filtered at the heliosphere boundary (Izmodenov et al. 1999; Baranov et al. 1991). The resulting relative abundances and velocity distributions of different neutral atoms in the heliosphere are different from original interstellar abundances and velocity distributions. H atoms effectively charge-exchange with plasma protons everywhere from the VLISM to the inner heliosphere, creating different H atom populations with different properties, e.g., the $12{,}000~^{\circ}\text{K}$ warm and 22 km/s slow H atoms in the hydrogen wall; the $\sim100{,}000~^{\circ}\text{K}$ hot H in the heliosheath and in the supersonic solar wind region (Quémerais and Izmodenov 2002; Heerikhuisen et al. 2016). Similarly, secondary helium and oxygen populations are also created (Bzowski et al. 2017; Park et al. 2019). The charge-exchange process leads to solar wind deceleration, especially beyond 30 au, which was confirmed by Voyager 2 Plasma Science (PLS) and New Horizons Solar Wind Around Pluto (SWAP) measurements (Elliott et al. 2019; Richardson et al. 1995; Wang et al. 2000). H atoms have a mean free path comparable to the size of the heliosphere, leading to an essentially non-Maxwellian H distribution function. The properties of H atoms in the heliosphere are controlled by the charge-exchange coupling with plasma, variations of the solar radiation pressure, and ionization due to extreme ultraviolet (EUV) photons and electron impact. These ionization processes create a cavity void of neutral hydrogen atoms close to the Sun, with a cavity size of $\sim4~\text{au}$ evolving with an amplitude of $\sim1~\text{au}$ during the solar cycle (Rucinski and Bzowski 1995; Sokół et al. 2019a; Quémerais et al. 2006b). Physical processes shaping the distribution of interstellar atoms in the heliosphere as well as their dependence on the solar cycle and VLISM conditions are fundamental to the formation of the entire heliosphere but are currently very poorly known.

### The Elusive Source of Anomalous Cosmic Rays

Anomalous cosmic rays (ACRs) (Hovestadt et al. 1973; McDonald et al. 1974) are likely produced from interstellar PUIs (Fisk et al. 1974) that are accelerated to energies of tens to hundreds of MeV/nuc (Geiss et al. 2006). Contrary to expectations, the Voyager mission did not observe a peak in ACR intensity across the TS. Instead, the ACR intensities continued to increase as the Voyagers traversed deeper into the heliosheath, indicating the importance of other, possibly remote, acceleration mechanisms. While several explanations emerged including acceleration at the flanks of the TS (McComas and Schwadron 2006), by compressive turbulence in the heliosheath (Fisk and Gloeckler 2009, 2012), by magnetic reconnection near the HP (Drake et al. 2010), and by small-scale flux ropes in the heliosheath (Zhao et al. 2019), the sources of ACRs remain elusive. To determine the energization pathway of ACRs and determine their source and relation to singly charged PUIs, measurements of protons, He, Li-Be-B, C, N, O, Ne, and other heavy ions from 100s of keV to $\sim100~\text{MeV/nuc}$ as well as their anisotropies are required. It must be stressed that composition is key for next-generation discoveries pertaining to ACRs because potential acceleration mechanisms, like diffusive shock acceleration, first order Fermi acceleration, and reconnection- and turbulence-driven acceleration are all mass dependent (e.g., Decker et al. 2005; Drake et al. 2006; Turner et al. 2018; Ergun et al. 2020). Understanding ACR acceleration is critical to a wide range of topics considering that ACRs contribute $\sim20\%$ of the thermal pressure in the heliosheath (e.g., Rankin et al. 2019a) and may be an important contribution to the seed population of higher-energy GCRs accelerated elsewhere in the galaxy. Better understanding of ACR sources and acceleration is also important to exoplanetary physics and the search for life in the universe because exoplanetary researchers typically only consider GCRs in energy input for atmospheric chemistry, but in some stellar systems with particularly efficient ACR acceleration, ACRs might dominate and contribute significantly to atmospheric chemistry in other astrospheres.

### The Porous Heliopause

When the Voyager mission finally crossed the HP (Burlaga et al. 2019; Krimigis et al. 2019), it did not encounter the theoretically expected sharp discontinuity separating the solar wind plasma and the VLISM plasma. Shortly after the crossing, the Plasma Wave Subsystem (PWS) on board Voyager 1 detected electron plasma oscillation at a frequency consistent with an electron density of $0.08~\text{cm}^{-3}$, which is very close to the expected value in the VLISM. However, Voyager discovered a region with complex interactions between heliospheric energetic particles and particles coming from interstellar space and magnetic fields of different origins. The two crossings of the HP share many similarities but also show some striking differences (Krimigis et al. 2019). For both crossings, inside the heliosphere there is a region of increased intensities of GCRs of similar spatial scale around 1 au. However, Voyager 1 observed several episodes of enhanced GCR intensities right before the HP crossing that were absent with Voyager 2. The situation with the heliospheric ions appears to be similar. The most noticeable difference is the extent of the upstream region before the disappearance of solar material, 0.25 au for Voyager 1 and 0.6 au for Voyager 2.

A Voyager 1 (Krimigis et al. 2013) there appeared to be a “depletion region” where energetic particles of solar origin flow outward reminiscent of an interchange instability between the solar wind plasma and the interstellar plasma. Later, the Low-Energy Charged Particle (LECP) experiment on board Voyager 1 clearly showed an anti-sunward flow of energetic ions, or “leakage”, in its 40–139 keV channels up to at least 28 au beyond the HP (Dialynas et al. 2021). An apparent leakage of solar particles out of the heliosheath that extends beyond the HP has also been reported at Voyager 2 (Krimigis et al. 2019).

Perhaps the most confounding observations at the HP were the lack of any significant rotation of the magnetic field across the HP, in either the Voyager 1 or 2 observations (Burlaga and Ness 2016), despite their drastic separation. While ideas started to form to understand the magnetic topology and particle interaction at the HP, the physical processes near this boundary remain an open question. It is unknown whether magnetic reconnection, turbulence, or viscous boundary interactions are important along the HP and to what extent they are enabling the interaction between the heliosphere and VLISM. Furthermore, arguments exist that the increased electron densities implied by PWS are not the VLISM densities, but a region of compressed, cold solar wind, and hence that Voyager 1 and 2 are still in the heliosheath (Gloeckler and Fisk 2015; Fisk and Gloeckler 2022). This region of cold plasma would better explain the Energetic Neutral Atom (ENA) spectrum below 30 eV observed by IBEX-Lo (Fuselier et al. 2012).

Critical observations of the full particle distributions (including PUIs) and fields on both sides of the HP are required to answer the outstanding questions remaining from the Voyagers’ crossings.

### The Sun’s Dynamical Sphere of Influence

The Sun’s activity causes various types of evolving multi-scale structures in the solar wind, from long-lived corotating interaction regions (CIRs) to more transient but more extreme events such as coronal mass ejections (CMEs). The solar wind dynamic pressure changes roughly by a factor of two from solar minimum to solar maximum and can vary by over two orders of magnitude from average conditions to those in transient phenomena such as CMEs. As structures in the solar wind propagate outward from the Sun, they evolve, merge, and interact with each other and the ambient solar wind. Voyager 1 and 2 provided the first in situ measurements of these structures in the outer heliosphere. In particular, Voyager observations in the heliosheath showed highly variable plasma flows indicating effects of solar variations extend from the Sun to the heliosphere boundaries. The Cassini (Krimigis et al. 2004) and IBEX (McComas et al. 2009b) missions mapped the ENA intensities across the sky for an entire solar cycle (Dialynas et al. 2017; McComas et al. 2017, 2019). The ENA images show substantial variations from solar minimum to maximum and responses to variations in solar wind dynamic pressure, demonstrating that the Sun’s activity drives the global response of the entire heliosphere and its interaction with the VLISM.

State-of-the-art simulations have demonstrated that effects of the solar cycle strongly influence the TS and HP locations and flows in the heliosheath (Baranov and Zaitsev 1998; Zank 1999; Scherer and Fahr 2003; Zank and Müller 2003; Izmodenov et al. 2005, 2008; Pogorelov et al. 2009). Models suggest that the TS reflects variations in the solar wind dynamic pressure observed at 1 au in about 1 year and that the TS position in the nose direction can fluctuate by 7 au about its mean value (Washimi et al. 2011), which was verified with direct measurements obtained by Voyager 1 and 2 (Krimigis et al. 2019). The boundaries of the heliosphere are constantly in motion. Despite the fact that Voyagers 1 and 2 crossed the HP under very different solar-cycle conditions and in different locations, the crossing distances are very similar, raising a question about how the HP responds to solar wind dynamic pressure changes. Although Krimigis et al. (2019) showed from Voyager measurements that the HP location is not directly sensitive to variations in solar wind dynamic pressure, models still predict several au displacements (Izmodenov et al. 2008). How multi-scale solar wind structures propagate and evolve in the outer heliosphere, what plasma flows they cause in the heliosheath, and how locations of boundaries change because of pressure pulses, shocks, and waves in the solar wind are open questions.

**Fig. 6:**
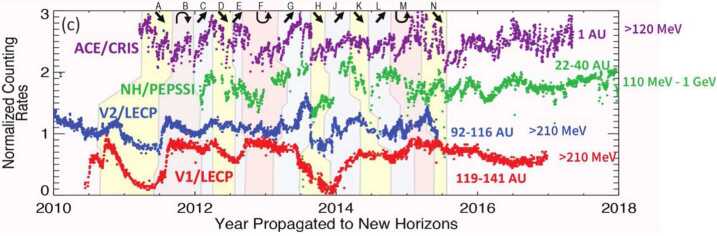
The variability of cosmic rays penetrating the heliosphere is a complex interplay between solar disturbances propagating through the heliosphere and even well beyond the HP. Daily averages of $\geq100~\text{MeV}$ proton rates are plotted from ACE, New Horizons to Voyager 1 and 2, and propagated to the position of New Horizons. The letters and respective colored regions mark different events with arrows denoting direction of variations. Adapted from Hill et al. (2020)

Voyager 1 and 2 unexpectedly discovered shocks and pressure waves beyond the HP in the VLISM (Burlaga et al. 2013; Gurnett and Kurth 2019) resulting from a complex propagation throughout the heliosphere that impact the global GCR flux (Fig. 6). Voyager 1 magnetic field data beyond the HP show a time interval (2014.6–2015.4) with 28-day oscillations in the magnetic field (Burlaga and Ness 2016) indicating a possible relationship with CIRs in the solar wind having periodicity of the solar rotation, directly indicating that the Sun influences this region. However, the origin of these oscillations is not fully understood. Simulations of shock evolution from the Sun to the VLISM show good agreement with the large-scale shocks observed by Voyager 1 up to mid-2016, suggesting that ICMEs play a critical role in the propagation of shocks (Kim et al. 2017). The properties of the broad and weak VLISM shocks observed by Voyager are surprisingly different from shocks in the heliosphere. The VLISM is a much colder and denser plasma than the heliosphere, which we have extensively explored with different missions. Thus, the very different physics of the VLISM affects the properties of shocks and turbulence in this region. Our understanding of the VLISM dynamics, drivers for shocks and waves, as well as their properties and evolution in the VLISM is very limited. With the available Voyager 1 data, however, models have revealed that the VLISM shocks are largely collisional, unlike shocks in the heliosphere, and are governed by thermal proton collisions (Mostafavi and Zank 2018a).

### The Global Manifestation of the Heliosphere

**Fig. 7:**
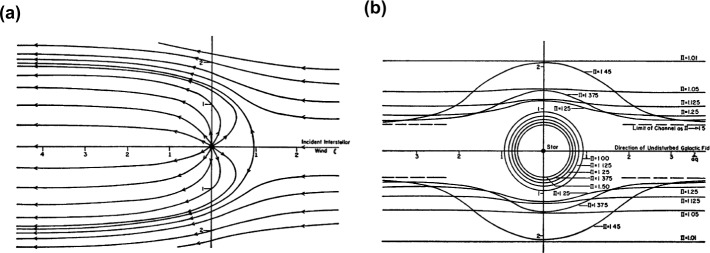
The results of Parker’s “rough calculation” of two extreme cases of the interaction between a stellar magnetic field with its surrounding interstellar magnetic field (Parker 1961). (**a**) A comet-like cavity as result of a relatively weak interstellar magnetic field, and, (**b**) A bubble-like cavity with a cylindrical channel extending from each stellar pole resulting from a strong magnetic field

The shape of the heliosphere, represented by the extent of the heliosheath out to the HP, is the most important constraint on the physics of the global interaction with the VLISM. Depending on the relation between the solar and interstellar magnetic field, Parker (1961) proposed two extreme cases of stellar interactions (Fig. 7a–b): A comet-like shape, in the case where the interstellar magnetic field pressure is weak compared to the LISM flow pressure and a spherical shape with a cylindrical channel extending from each pole in the case where the interstellar magnetic field pressure dominates over the flow pressure. Although constituting a “rough calculation of the cavity”, the resulting two extreme cases are still central to the scientific debate today.

Voyager 1 and 2 crossed the HP in the nose hemisphere in 2012 and 2018, respectively, revealing surprisingly similar distances from the Sun to the HP, 121 au for Voyager 1 and 119 au for Voyager 2. This similarity is despite the fact that the two Voyager spacecraft were separated $60^{\circ}$ in latitude and 170 au in distance and the crossings occurred in different solar-cycle conditions. Imaging of the global interaction in energetic neutral atoms (ENAs) from the Interstellar Boundary Explorer (IBEX) (0.01–6 keV; e.g. Galli et al. 2022) and Cassini/Ion and Neutral Camera (INCA) (5–55 keV; e.g. Dialynas et al. 2022) missions have provided a unique opportunity to gain insights into the global heliosphere shape and size. ENA observations on IBEX and Cassini in different energy ranges revealed completely unexpected emission features in the sky: the IBEX ENA ribbon (McComas et al. 2009a) and the Cassini ENA belt (Krimigis et al. 2009). Explanations of the generation mechanisms behind these global features still remain somewhat inconclusive. The “secondary charge-exchange” hypothesis for the origin of the IBEX ribbon at 6 keV has largely been accepted by the IBEX team (Zirnstein et al. 2015; McComas et al. 2017, 2020; Schwadron et al. 2018a; Swaczyna et al. 2022a), while the $>5.2~\text{keV}$ ENAs from the Belt are shown to be formed by charge-exchange interactions in the heliosheath (Krimigis et al. 2009; Dialynas et al. 2022).

By applying a “sounding technique” using energy dependent propagation of ENAs and spectral analyses, IBEX-Hi and Cassini/INCA ENA observations have been used to constrain the heliospheric shape from their vantage points deep inside the heliosphere. The first three years of IBEX-Hi data seem to suggest an intermediate case with a “heliotail” between the two Parker models (McComas et al. 2013). Although no precise length of a heliotail could be derived from these data, IBEX-Hi images indicated two tailward lobe regions with steeper energy spectra suggestive of the fast solar wind originating from the solar poles. More recent estimates using the sounding technique on the extensive IBEX-Hi data set indicates an ENA source region extending to at least 380 au, where the HP must be farther out (Reisenfeld et al. 2021). Using the Cassini/INCA and similar sounding technique, Dialynas et al. (2017) reported a roughly spherical heliosphere in all directions. Although not entirely clear, the differences between the IBEX and Cassini results may be attributed to the different energy ranges of the two data sets or differing interpretations of the data sets (e.g., Schwadron and Bzowski 2018), but it has to be kept in mind that the uncertainties of both results are still relatively significant due to the fact that both vantage points are deep inside the heliosphere. NASA’s upcoming Interstellar Mapping and Acceleration Probe (IMAP) mission (McComas et al. 2018) will provide a leap in imaging capabilities, resolution and sensitivity from 1 au that is certain to significantly further our understanding of the global interaction.

**Fig. 8:**
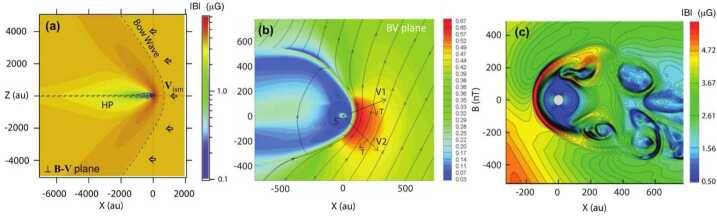
(**a**) Model by Zhang et al. (2020) showing a significant heliotail extending several 1000 au. (**b**) Model by Izmodenov and Alexashov (2020) showing a comet-like shape. (**c**) Model by Opher et al. (2020) showing a croissant-like heliosphere with turbulent jets

None of the existing state-of-the-art models of the global interaction of the solar wind with the VLISM fully agree with current observational estimates of the shape of the heliosphere. Figure 8a shows a comet-like heliosphere with a long, turbulent heliotail extending several 1000 au which might explain the spatial (Zhang et al. 2020; Pogorelov et al. 2015) anisotropies of TeV GCRs observed from Earth-based measurements (Amenomori et al. 2006). Figure 8b also shows a comet-like heliosphere using the magnetic field measurements obtained by Voyager beyond the HP (Izmodenov and Alexashov 2020). Lastly, Fig. 8c shows a croissant-shaped heliosphere with two “jets” folded back (Opher et al. 2020) corresponding to the “cylindrical channel extending from each pole” in the work by Parker (1961). While this model produces a more bubble-like heliosphere, it still makes a’priori assumptions on the treatment of neutrals and PUIs that are not yet self-consistent nor fully understood.

A fast trajectory through the heliosphere into the VLISM of a future Interstellar Probe offers a unique platform for remote ENA observations and in situ measurements within the emission source region. ENA observations of the ribbon, the globally distributed flux, the belt and global structure along a changing vantage point would provide important constraints on physical mechanisms and the overall heliospheric shape. In-situ measurements of the particle distributions and fields within the source region of the ribbon and belt, such as pitch-angle distributions and plasma flows, would provide a direct insight to directly determine their generation mechanisms, and more importantly their link to the global heliosphere structure and interaction with the VLISM. Starting at around 200 au, the external vantage point would offer the first unique ENA image from the outside. In particular, ENA images at 80 keV of hydrogen from a trajectory $40^{\circ}$ or more off the heliosphere nose direction would be able to reveal the potential two-jet structure predicted by Opher et al. (2020). See Sect. 5 for more details. This external vantage point would therefore provide the strongest constraint to global physical models, the shape of the heliosphere, and its dynamic response to solar variability.

## Discoveries Beyond

The known mysteries uncovered by the Voyager mission and others summarized above must be solved to make progress in understanding the physical interactions with the VLISM. Several other unknown mysteries still await to be uncovered in the regions already visited by Voyager. However, the most significant discoveries yet lie likely beyond the regions explored by Voyager. As is clear from their further exploration the Sun’s sphere of influence extends well beyond the HP. The Voyager spacecrafts are projected to remain operational until at least 2030 (S. Dodd, Personal Communication) corresponding to a heliocentric distance of about 185 au for Voyager 1 and 155 au for Voyager 2. It is also becoming very likely that New Horizons will have sufficient power to become the third operational spacecraft to cross the TS well into the heliosheath (S.A. Stern, Personal Communication).

Many features beyond the current in-situ exploration have been predicted or inferred from remote observations, such as the bow wave and the hydrogen wall. However, a large part of interstellar matter has no access to the heliosphere and therefore remains a completely unexplored territory, including interstellar dust (ISD) grains, $\leq2~\text{GeV}$ GCRs, interstellar plasma and of course, the pristine interstellar magnetic field. The knowledge of the surrounding VLISM, such as the flow, temperature, and elemental and isotopic composition of gas and plasma remains very scarce. All of our knowledge about the LIC and other neighboring clouds is based on line-of-sight absorption spectra to the nearest stars. Although there is now growing evidence that the heliosphere is about to leave the LIC and enter the G-cloud, very little is known about their detailed properties.

In this section we summarize the limited knowledge available from the current sphere of exploration defined by the Voyager mission, and outline the potential consequences for the heliosphere.

### The Existence and Nature of a Bow Wave

Depending on a star’s speed of motion in the ISM and properties of the interstellar gas itself, a bow shock may form ahead of the astrosphere. Several such bow shocks are prominent ultraviolet (UV) and infrared observations, but are all around astrospheres very different than our own (Cox et al. 2012; Baalmann et al. 2020; Peri et al. 2015; Kobulnicky et al. 2016; Katushkina et al. 2018).

State-of-the-art physics-based multicomponent models of the solar wind interaction with the VLISM predicted an existence of the bow shock ahead of the heliosphere, a sharp transition where interstellar plasma flow becomes subsonic (Izmodenov 2009; Zank et al. 2009; Fahr and Siewert 2007a). The VLISM flow relative to the Sun is supersonic, but it can be below the propagation speed of fast magnetosonic modes, depending on the unknown magnetic field in the VLISM. In the case of a strong interstellar magnetic field, formation of a fast-mode bow shock is not possible. If the angle between magnetic field direction and velocity is small, then a formation of a slow model bow shock remains possible (Florinski et al. 2004; Pogorelov et al. 2011; Chalov et al. 2010; Zieger et al. 2013; Fahr et al. 2007b). Heating of the VLISM plasma induced by charge exchange of incoming ENAs from the heliosphere may result in increased fast magnetosonic speed in the VLISM and weakening or even elimination of the bow shock structure (Pogorelov et al. 2017; Zank et al. 2013). In this case, the broad region of slowed down and piled up VLISM plasma forms is called a bow wave. IBEX observations have been used to derive the relative velocity vector of the heliosphere through the VLISM that indicate that the speed is slower than the propagation of the fast magnetosonic mode and therefore likely rules out a bow shock (McComas et al. 2012). Although from current values it appears that a bow wave structure is expected, only in situ measurements will provide definitive determination on the nature and existence of a potential bow wave and how this structure may depend on changing VLISM conditions. Such detailed understanding will serve as important ground truth for understanding the physics of bow shock structure around other stars that in turn are used for probing their stellar wind and VLISM properties.

### Hydrogen Wall

The hydrogen wall (H-wall) is a pileup of interstellar hydrogen beyond the heliosphere boundary created by H atoms originating in a charge exchange between “pristine” interstellar H and slowed down and heated interstellar plasma flowing around the heliosphere. Predicted by models of the outer heliosphere (Baranov and Malama 1993; Gruntman et al. 2001; Zank et al. 2013), the H-wall is thought to be located near 300 au and may extend outward to 400–600 au. Analogous to the H-wall, there may also exist an oxygen wall (O-wall) of secondary interstellar oxygen atoms that originated in a charge exchange between oxygen ions and hydrogen (Izmodenov et al. 2004). Heliosphere H-wall absorption was discovered for the first time by Linsky and Wood (1996) in the Lyman-$\alpha $ spectra toward alpha-Centauri measured by the Hubble Space Telescope/Goddard High Resolution Spectrograph (GHRS). The presence of a hydrogen layer near the heliosphere boundary is also suggested by the Voyager/Ultraviolet Spectrometer (UVS) Lyman-$\alpha $ data (Quémerais et al. 2000b; Katushkina et al. 2017). The Hubble Space Telescope found evidence of an H-wall around other stars, indicating that an H-wall is a common phenomenon for astrospheres. The most relevant example is the H-wall detected by Wood et al. (2004) around alpha-Centauri A and B.

Given the governing importance of neutral interactions with the heliosphere, characterizing the H-wall (and other species’ walls) remains one of the crucial investigations to be conducted by a future mission. In-situ measurements of properties, such as location of peak density, spatial extension, shape, and density profile of hydrogen (and oxygen) in the H-wall (O-wall) compared to the unperturbed VLISM would impose among the strongest and definitive constraints on global models.

### Unshielded Galactic Cosmic Rays

GCRs in the 1 MeV/nuc to 1 GeV/nuc range are deflected by the heliosphere, and thus $>75\%$ of GCRs never reach the inner solar system where they otherwise could affect the chemical evolution of atmospheres. Therefore, it is important for general planetary habitability studies to understand how an astrosphere shields its planetary system from GCRs. A vantage point well into the pristine VLISM, where our Sun no longer has direct influence, would provide direct access to spectra of GCRs unperturbed by the heliosphere and therefore would provide further insight into their source and interaction with the galaxy. Critically, the Voyager cosmic ray instruments could not resolve isotopic masses of measured cosmic rays, yet as outlined (Mewaldt 2013; Wiedenbeck 2013; Wiedenbeck et al. 2007, and references therein), measurements of rare and unstable cosmic ray isotopes can be used to answer questions pertaining to cosmic ray source regions via spallation and direct acceleration, galactic escape rates, and solar modulation. These open questions and unobserved species of GCRs in the VLISM are of importance not only to heliophysics and the nature of particle acceleration and consequences of GCRs in the heliosphere, but also to astrophysics and the nature of the universe itself. Observations of particularly rare GCR isotopes, GCR electrons, and antimatter in the VLISM can even shed light on and further constrain cosmological models. For example, GCR positrons may form through pair annihilation of weakly interacting massive particles believed to be a major constituent of dark matter that were created in the early universe (Di Mauro et al. 2016; Boudaud et al. 2017). While high-energy telescopes have measured positrons above several GeV that penetrate the heliosphere, observations of the low-energy component unperturbed by the heliosphere are required to firmly establish the dark-matter hypothesis. Furthermore, recent work finds an unexpected excess of Fe at energies near a GeV/nucleon, consistent with the terrestrial records of ^60^Fe pointing to recent supernova activity in the Local Bubble (Boschini et al. 2021).

Lithium, beryllium, and boron (Li, Be, and B) have very low nuclear binding energies and are not produced in any significant abundance by our Sun (and stars like it). In the ISM, however, Li, Be, and B are produced by cosmic ray spallation, and Li has an additional source in the deaths of certain low-mass stars. At cosmic ray energies, the abundance of Li, Be, and B is comparable (same order of magnitude) to that of C, N, and O, which is entirely different than the relative abundances within the heliosphere (e.g., Wiedenbeck et al. 2007). The heliospheric relative abundances of Li, Be, and B are four to six orders of magnitude lower compared to their relative abundances in the ISM. Their spectra at lower energies ($\sim50~\text{MeV/nuc}$) in the VLISM provide important information on their sources (spallation versus stellar) and remain unknown due to the lack of measurements.

### Interstellar Dust Grains: Messengers of Galactic and Stellar Evolution

The VLISM consists of material in multiple hot, warm, and cold phases, each of which is characterized by different temperatures, densities, and stages of ionization—both atomic and molecular—as well as ISD grains. These are the condensed phases of the ISM, transporting the heavy elements produced by stellar nucleosynthesis through the different ISM phases (Draine 2009). Although representing only $\sim1\%$ of the mass of the ISM, ISD grains contribute significantly to the different evolutionary processes of the galaxy. They are the building blocks of new stellar and planetary systems that form from collapses of cold molecular clouds. Dust condensation from gaseous heavy elements occurs both in certain circumstellar environments as well as in protostellar nebulae. ISD grains ensure the transport and mixing of heavy elements across the different phases of the ISM, where they undergo multiple cycles of formation and destruction (Zhukovska et al. 2008). Any model describing galactic chemical evolution must therefore take the grain life cycles through the ISM into consideration. A direct in situ characterization of the ISD grains in the warm gas and dust phase surrounding the solar system, the LISM, and their interaction with the gas phase therefore enables an understanding of the true nature of the current building blocks of planetary systems in our galaxy.

Properties of ISD in the heliosphere are affected by deflection and filtration processes at the heliosphere boundary and effects near the Sun, such as gravity, radiation pressure, solar wind drag, and electromagnetic forces (Landgraf et al. 1999). Many authors have simulated the deflection of dust particles of various sizes in the heliospheric interface region (Landgraf 2000; Slavin et al. 2012; Sterken et al. 2013, 2012; Godenko and Izmodenov 2021; Alexashov et al. 2016). Simulations predict that dust particles less than about 20 nm do not penetrate into the heliosphere, but flow around the HP affected by the interstellar magnetic field. Distribution of ISD particles of any size inside the heliosphere can be very inhomogeneous in space (Godenko and Izmodenov 2021; Landgraf 2000; Sterken 2012; Sterken et al. 2013; Slavin et al. 2012) with large regions of density enhancement due to the interaction of the changing polarity of the solar current sheet. These authors explored effects on the ISD in the heliosphere of the time-dependent heliospheric magnetic field with the 22-year periodic changes of the heliospheric current sheet inclination and the 25-day rotation of the Sun and showed focusing and defocusing of dust over a solar cycle. We are just beginning to explore the effects of ISD and synergies with heliosphere science. Dust and plasma go hand in hand because of the coupling of the dust with magnetic fields and charging of the dust by the plasma. Properties of the ISD inside and outside the heliosphere, deflection and filtration processes, and possible effects of the dust on PUI production in the heliosphere remain open questions.

### Properties of the Changing Interstellar Cloud Neighbourhood

The VLISM beyond where the Voyager mission has explored is a completely new territory for discovery. We have only a very crude understanding of the VLISM environment inferred from in situ measurements inside the heliosphere of interstellar helium, PUIs, ENAs, remote observations of solar backscattered Lyman-$\alpha $ emission, and absorption line spectroscopy in the lines of sight of stars. Almost all of the key driving parameters that significantly affect the heliosphere have never been measured directly in the unperturbed VLISM. Only very few of the properties have been measured directly by the Voyager’s limited payload, and only in the region still affected by the Sun.

The interstellar magnetic field upstream at the HP (in the compressed region) has been directly measured only by Voyager, and calculated accurately from the pressure balance at the HP by combining remotely sensed ENAs and in-situ ions (e.g. Dialynas et al. 2020, 2019), but appears to still be near the direction of that of the Parker spiral (Burlaga and Ness 2016; Burlaga et al. 2019). Zirnstein et al. (2016) used a magnetohydrodynamic (MHD) model coupled with a kinetic treatment of neutral hydrogen to obtain a best fit between the simulated and observed IBEX ribbon using the magnetic field at infinity as the free parameter. The best fit indicates a VLISM field at a 1000 au of magnitude $(0.293\pm0.008)~\text{nT}$ and a direction of $227.28^{\circ} \pm 0.69^{\circ}$ ecliptic lon, $34.62^{\circ}\pm0.45^{\circ}$ ecliptic lat. The resulting modeled magnetic field magnitude and direction at Voyager 1 was consistent with measurements, and the field direction at infinity was offset $8.3^{\circ}$ from the center of the ribbon. Izmodenov and Alexashov (2020) used a kinetic MHD model constrained by the Voyager 1 and 2 magnetic field measurements to estimate a field magnitude 0.37–0.38 nT at 400–500 au with a direction of $\sim125^{\circ}$ longitude and $\sim37^{\circ}$ latitude in heliographic coordinates, or approximately $205^{\circ}$ elon, $43^{\circ}$ elat.

Of the many plasma and neutral density estimates, only the total electron density has been measured, by the Plasma Wave Subsystem (PWS) on board Voyager 1 and 2 (Gurnett and Kurth 2019; Kurth and Gurnett 2020). Other estimates include derivations using PUI measurements from Ulysses and ACE (Gloeckler and Geiss 2004), ENA and in-situ ions from Cassini, the Voyagers (Dialynas et al. 2019), and from New Horizons (Swaczyna et al. 2020), absorption spectra to the nearest stars obtained by the Hubble Space Telescope (HST) (Redfield and Linsky 2008), and inferences from models constrained by direct measurements (Slavin and Frisch 2008; Zank et al. 2013). Lastly, interstellar flow vectors and temperatures have been estimated from Ulysses He measurements (Witte et al. 2004; Wood et al. 2015), HST absorption spectra (Redfield and Linsky 2008; Linsky et al. 2019), IBEX (Bzowski et al. 2012; Möbius et al. 2012; McComas et al. 2015; Swaczyna et al. 2022b; Schwadron et al. 2021), and SOHO and Prognoz (Lallement et al. 2004).

**Fig. 9:**
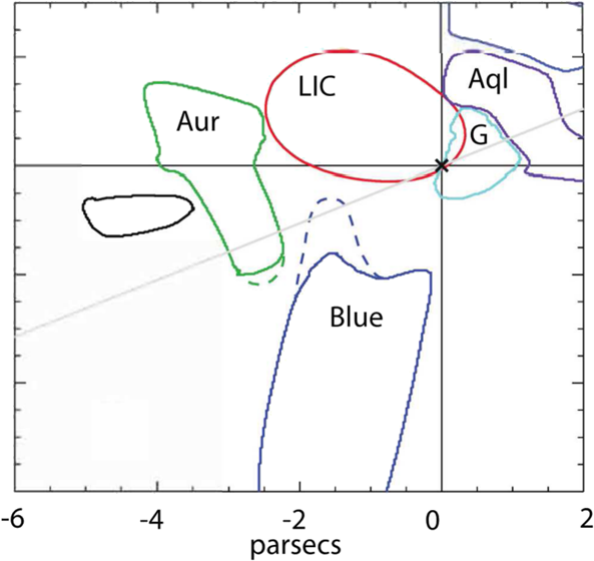
The Sun is estimated to be very near the edge of the LIC and may be in the completely new environment of the G-cloud within several 1000’s years (Linsky et al. 2022)

The current knowledge of the large-scale properties of our interstellar cloud neighborhood come from absorption spectra along LOSs to the nearest stars (Redfield and Linsky 2008; Frisch 2009; Linsky and Redfield 2021; Linsky et al. 2019). Some 60,000 years ago, the Sun entered the LIC and is now at the very edge of it, or is already in a transition region towards the G-cloud (Fig. 9). Over the course of the journey around the galactic core the heliosphere has traversed clouds with very different properties that have dramatically affected its size and interaction, that in turn have drastically altered the exposure of the inner solar system to interstellar GCRs, dust, gas and plasma (see Sect. 3.6 for more details). It is now also becoming clear from the many LOS measurements that the upper limit on length scales in the interstellar clouds are as little as several 1000’s of au (Linsky et al. 2022). By virtue of the relative motion of the VLISM, this may make it possible to assess the gradients in the LIC with a mission to the VLISM out to multiple hundreds of au.

It is commonly assumed that the VLISM encountering the heliosphere has been constant over the decades of measurements. Frisch et al. (2013) argued that different values of VLISM flow direction and speed obtained by IBEX-Lo (Fuselier et al. 2012; Möbius et al. 2012) is a true change over the decades since past measurements (Witte et al. 2004; Möbius et al. 2004) and may be due to non-thermal or Alfvenic turbulence in the LIC. However, a debate is still ongoing whether or not such changes can be confirmed or denied from observations (Lallement and Bertaux 2014; Frisch et al. 2015). Swaczyna et al. (2022b) analyzed 12 years of IBEX-Lo observations and estimated an upper limit of this shift to be three times smaller than originally reported by Frisch et al. (2013).

The role of nonthermal ions continues to be a complete unknown in the VLISM, but they may play a decisive role in the structure of the LIC (and others like it) and in the entire force balance with the heliosphere (Linsky et al. 2019). There is no reason to believe that the plasma in the VLISM is in thermal or ionization equilibrium or that nonthermal particles do not dominate the ionization and total pressure. Chassefiere et al. (1986) showed that the timescales for ionization and recombination are on the order of $10^{7}$ years, but supernovae in the nearby Scorpio–Centaurus Association have occurred as recently as a few million years ago, and their shock waves could have produced high ionization in the VLISM that is still recombining. New models of the velocity distribution of plasma in the outer heliosphere are beginning to include nonthermal components through the use of kappa functions (Vasyliunas 1968) that differ from Maxwell–Boltzmann velocity distributions by including high-velocity tails (Swaczyna et al. 2019). Nonthermal ions in the VLISM (Gloeckler et al. 1997; Izmodenov et al. 2001) have also been hypothesized to originate from the ionized component of the neutral solar wind that is transported across the HP into the VLISM, where it is ionized through charge exchange and possibly by electron impact including possible turbulent heating in the VLISM.

Magnetic fields will also be important in shaping the morphology of partially ionized clouds if the magnetic pressure exceeds the gas pressure in the VLISM clouds. The local interstellar magnetic field strength of $0.293\pm0.008~\text{nT}$ estimated by Zirnstein et al. (2016) is close to the equipartition with the gas pressure in the LIC, $\text{P}_{\mathrm{gas}}/\text{k}\approx 2500~\text{cm}^{-3}\,\text{K}$. If the field strength in the unperturbed VLISM is larger, such as the one derived by Dialynas et al. (2019) from Voyager 2 charged-particle measurements in the HP and from Cassini data, this would dominate the gas pressure and thereby shape the partially ionized VLISM clouds.

It is unclear whether or not the LIC has solar abundances. Although ACR measurements of isotope ratios may suggest similar values (Leske 2000), the source of the ACRs is still elusive. Furthermore, solar isotopic ratios are also attached with some uncertainty (Gloeckler and Geiss 2007). Slavin and Frisch (2006) found a surprising overabundance of C in the LIC, which they speculated could be due to the destruction of C bearing dust grains by an interstellar shock in the past.

The first direct insights into the physical processes responsible for the LIC and the Local Bubble can only be obtained by a direct characterization of the unperturbed VLISM (Linsky and Redfield 2021).

### The Evolutionary Journey of the Solar System

There is overwhelming geological evidence from ^60^Fe and ^244^Pu isotopes that Earth was in direct contact with the ISM 2–3 million years ago. ^60^Fe has a half-life of 2.6 million years and is not naturally produced on Earth. ^60^Fe is predominantly produced in the winds of massive stars and in supernova explosions. Evidence of deposition of extraterrestrial ^60^Fe on Earth was found in deep sea sediments and ferromanganese crusts between 1.7–3.2 million years ago (Ma) (Wallner et al. 2016, 2021; Knie et al. 1999; Fitoussi et al. 2008), in Antarctic snow (Koll et al. 2019), and in lunar samples (Fimiani et al. 2016). The abundances were derived from new high precision accelerator mass spectrometry measurements. In addition, cosmic ray data assembled by the Advanced Composition Explorer (ACE) spacecraft measured the ^60^Fe abundance as well (Binns et al. 2016).

Studies have attributed the two peaks in ^60^Fe to multiple supernova explosions within 100 pc over the last 10 Myr that formed the local bubble (Fields et al. 2008) and brought ^244^Pu to Earth through supernova ejecta or encounter of the Solar system with clouds enriched with ^60^Fe dust.

Other studies suggested that nearby supernova explosions within $\sim10\text{--}20~\text{pc}$ could have produced the above isotopes (Fields et al. 2008). In particular, the heliosphere would shrink down to just beyond 1 au for a nearby supernova as close as 10 pc. This scenario requires fine tuning since this distance is very close to the so called “kill radius” of 8 pc, where extinction of all terrestrial life would have been triggered. For a supernova at a larger distance, there is a need to deliver ^60^Fe to Earth. It is also unclear whether traversing the shock width from a single supernova explosion would be consistent with the duration of 1.5 Myr inferred from ^60^Fe data.

The solar system neighborhood is not homogeneous. On parsec scales the solar system has been located inside a local super bubble (LB) (with size $\sim200~\text{pc}$) (Zucker et al. 2022) with a hydrogen density of $\sim0.005~\text{cm}^{-3}$ and temperature $T\sim10^{6}~^{\circ}\text{K}$. On smaller scales ($\sim15\text{--}20~\text{pc}$) several partially ionized clouds exist with hydrogen densities of $\sim0.1~\text{cm}^{-3}$ and $T\sim7000~^{\circ}\text{K}$ (Redfield and Linsky 2008; Linsky et al. 2022). The Sun moves with speed of 18 pc/Myr and it is clear that the solar system has traversed different regions of the local interstellar medium (ISM) during the past several million years that have affected its heliosphere.

It is known that different conditions affect the size and characteristics of heliosphere. Presently the heliosphere has a size of $\sim100~\text{au}$ and is located inside a partially ionized medium with a hydrogen density of $0.15~\text{cm}^{-3}$ and a temperature of $\sim6000~^{\circ}\text{K}$. Müller et al. (2006) showed that the heliosphere can shrink to a size of 23 au in an interstellar medium 100 times denser than today.

**Fig. 10:**
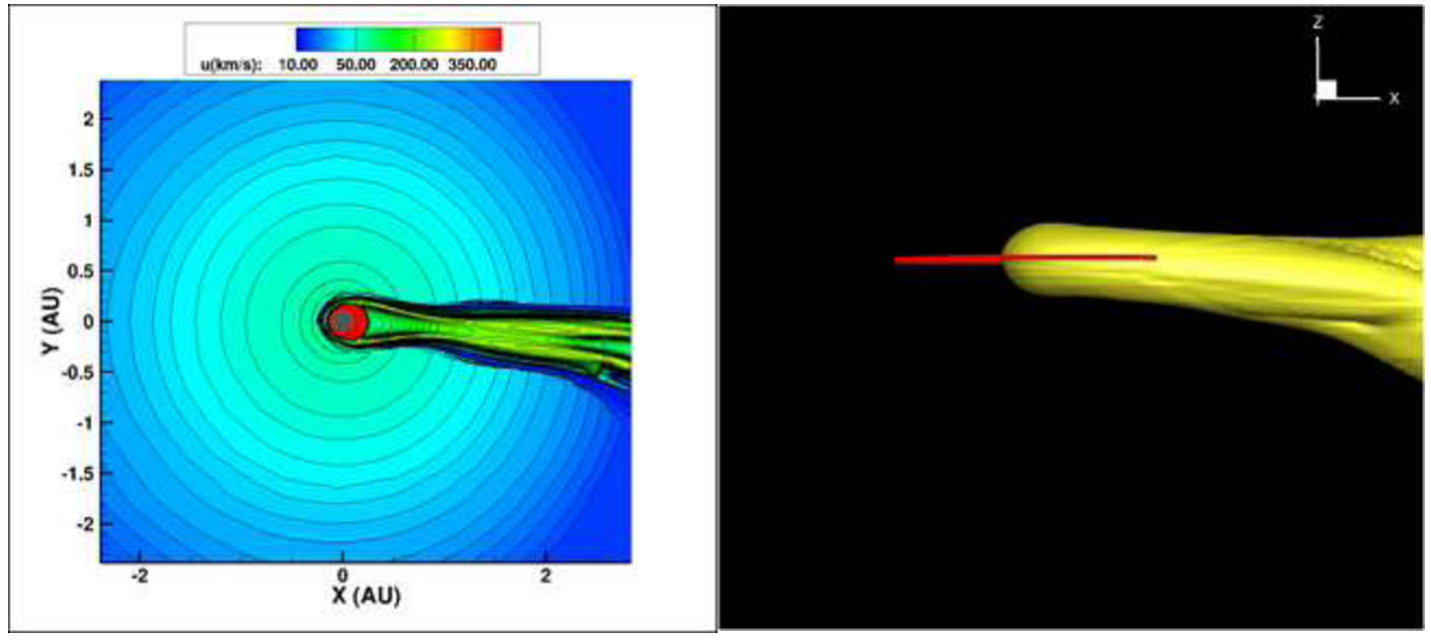
The Heliosphere 2 Myr ago. Panels are shown at the end of the simulation at 1.3 yr. Panel (**A**) is in the meridional plane at $\text{y}=0~\text{au}$. Contours shown are speed. The Heliosphere shrinks to 0.22 au at the nose, maintaining a long cometary shape and exposing all planets to the cold dense ISM material. Panel (**B**) show a 3D image of the heliosphere with two views. The trajectory of Earth is plotted in red. The iso-surface of the heliosphere is plotted at neutral density $n_{\mathrm{H}}=2000~\text{cm}^{-3}$

Opher and Loeb (2022) asked the question – what if the heliosphere had encountered a massive neighboring cloud that had compressed the heliosphere to the orbit of Earth and exposed the Earth directly to the interstellar medium? Such clouds in fact exist and one example is the Local Leo Cold Cloud (Peek et al. 2011) located between 23–45 pc from the Sun that has a hydrogen density of $\sim3000~\text{cm}^{-3}$ and a temperature of $20~^{\circ}\text{K}$ (Meyer et al. 2012). The Local Leo Cold Cloud could also be part of a Local Ribbon of Cold Clouds (Haud 2010). Opher and Loeb (2022) simulated the encounter of the heliosphere with a cold cloud (Opher et al. 2021, 2015) such as the Local Leo Cold Cloud (Meyer et al. 2006). The heliosphere shrunk to 0.22 au, a smaller than the Earth’s orbit around the Sun (Fig. 10). Earth was exposed to a neutral hydrogen density near $3000~\text{cm}^{-3}$. This H density could have had drastic effects on Earth’s climate, creating global ice sheets that can cool down the atmosphere (McKay and Thomas 1978; Yabushita 1997).

This scenario is consistent with isotope oxygen data measured in foraminifera in the sea floor (Zachos et al. 2001), mapping the paleo climate at the time when there had been a rapid cooling. There have been suggestions that increased climate fluctuations at that time had an impact on the human evolution (deMenocal 2011; National Research Council 2010; Potts and Faith 2015). If this is indeed the case, then the passage of the solar system through a cold cloud, consistent with the ^60^Fe data and with paleo-climate data, would have had an impact on human evolution as well.

## Outstanding Science Questions

An Interstellar Probe would transect the heliosphere from Earth to the VLISM to address the vast range of science only briefly described above. The outstanding science questions that could be answered by the pragmatic Interstellar Probe mission concept that was studied are summarized in Table 2. The complete tracing from these science questions to measurement, spacecraft and mission requirements is a complex, but critical exercise for any mission, and the resulting so-called Science Traceability Matrix from the Interstellar Probe Mission Concept Report can be found here.

## Cross-Divisional Opportunities

Understanding the origin and evolution of our solar system, and of planetary systems around other stars, are fundamental to achieving the science goals for the NASA Planetary Science and Astrophysics divisions. In addition to the example heliophysics baseline mission concept, an augmented Interstellar Probe concept has also been studied, but its technical details or trades are beyond the scope of this paper.

**Fig. 11:**
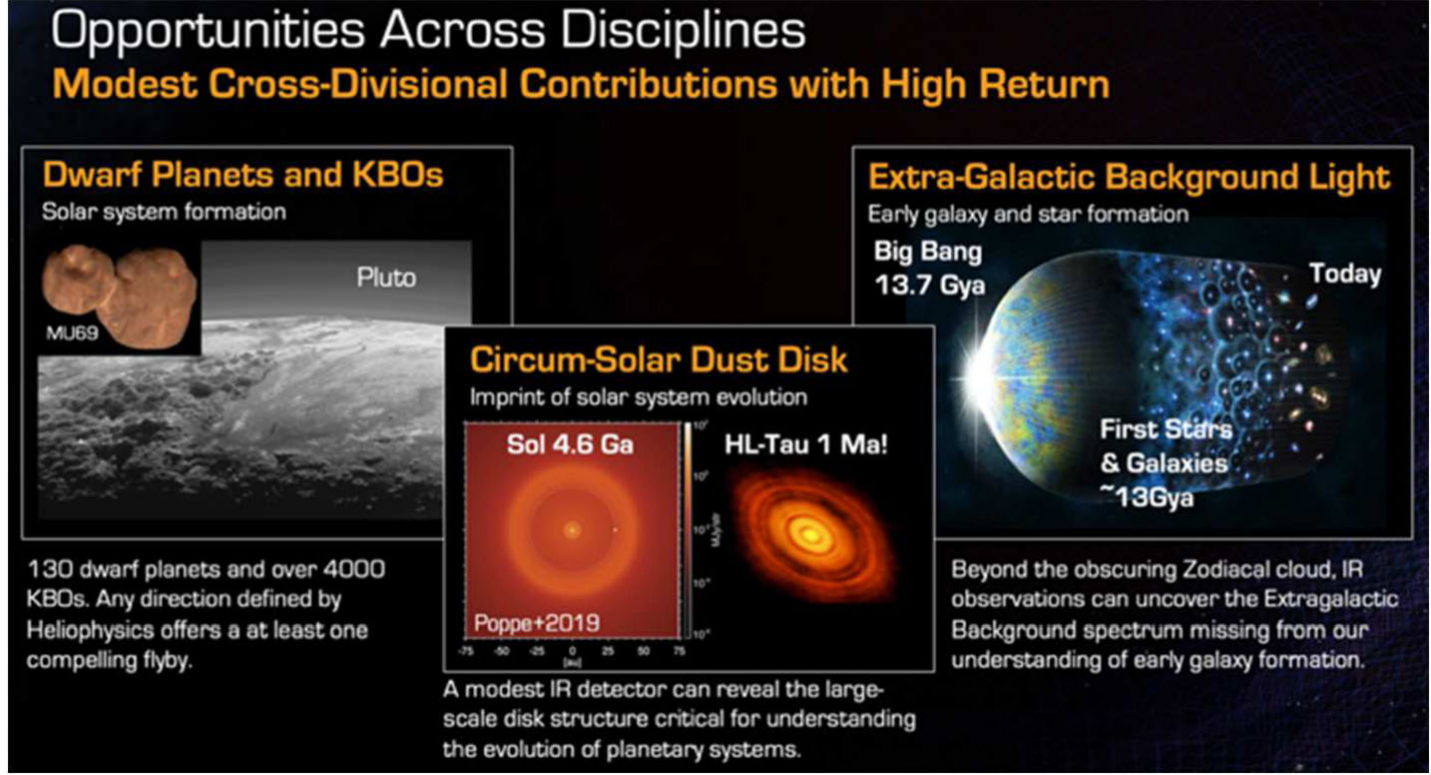
A wide range of unique, transformational science can be done from the Interstellar Probe spacecraft heading out of the solar system with modern purpose-built instrumentation, including close flybys of outer-solar-system planetesimals and dwarf planets, imaging of our solar system’s entire circumstellar debris disk and planets as exoplanets, and accurate measurement of the cosmic background light. Note: IR, infrared; KBO, Kuiper Belt object

An outward trajectory through the outer solar system would offer leaps in our understanding by taking direct measurements of small planetary bodies and of the dust before reaching the heliopause and even beyond the heliopause. Dwarf planets are defined here as round, planetary bodies larger than 400 km in diameter up to roughly the size of Pluto, which is 2377-km diameter (Nimmo et al. 2017). The number of currently known dwarf planets is estimated to be about $\sim130$ (http://web.gps.caltech.edu/~mbrown/dps.html) in the trans-Neptunian region and thus represent the largest category of planets, far outnumbering giant and terrestrial planets in the solar system. Many of these planets may be or may have been ocean worlds—targets of great astrobiological interest. One such dwarf planet is Orcus and its large moon Vanth (Fig. 11a) with neutral color and strong water-ice absorption features, along with an unidentified spectral feature that could be due to either ammonia hydrates or methane (Barucci et al. 2008; Carry et al. 2011; Delsanti et al. 2010; Fornasier et al. 2004). Detection of ammoniated species would make it the only TNO outside the Pluto system with such ices and could indicate past or present cryovolcanic episodes. Some have even suggested that the presence of NH_3_ and water ice provides evidence for a subsurface ocean (Hussmann et al. 2006). Geologic information obtained as part of a flyby would be useful for comparison to the surface of Charon and could even be used to look for short-timescale resurfacing processes. Active cryovolcanic eruptions or the detection of a magnetic field would be strong evidence for a subsurface ocean below Orcus’ crust.

Planetesimal belts and dusty debris disks around stars represent signposts of planetary formation. Their overall brightness provides information on the amount of sourcing planetesimal material, while asymmetries in the shape of the disk can be used to search for perturbing planets. The solar system is known to house two such belts, the inner Jupiter-family comet (JFC) and asteroid belt and the outer Edgeworth-Kuiper Belt (EKB), and at least one debris cloud, the zodiacal cloud, sourced by planetesimal collisions and comet evaporative sublimation. Close to Earth, the composition and the structure of our circumsolar dust disk are relatively well understood (Leinert et al. 1998; Kelsall et al. 1998; Rowan-Robinson and May 2013; Tsumura et al. 2013). However, beyond 1 au, the dust disk is poorly understood due to its obscuring properties for remote infrared observations and due to the lack of in-situ measurements. The only spacecraft to have flown dust measurement capability through the EKB are New Horizons (Piquette et al. 2019; Bernardoni et al. 2022) and the Voyagers via the Plasma Wave System (Gurnett et al. 1997). New estimates from the New Horizons results put the EKB disk mass at 30–40 times the inner disk mass (Poppe et al. 2019). Better understanding how much dust is produced in the EKB will improve our estimates of the total number of bodies in the belt, especially the smallest ones, and their dynamical collisional state.

Lack of knowledge of our own system is a major hindrance as we begin to probe the equivalent structures in exoplanetary systems (e.g., review by Hughes et al. 2018). Models indicate that there should be structures associated with Neptune and the EKB, to which we see many analogs in the circumstellar disks around other stars (Fig. 11b). We have virtually no understanding of how these disks compare to our own, where we can hope to study composition and small-scale structures directly. Observations probing interplanetary dust particle (IDP) emissions at a variety of wavelengths along different sight lines, as we pass through and emerge from the cloud, are necessary to develop a 3D understanding of the morphology of our own dust disk and to contrast it with those of exoplanetary systems.

Beyond the bulk of the “zodiacal” circumsolar dust cloud enveloping the Earth, past 10 au from the Sun, the outer solar system is a unique, quiet vantage point from which to observe the extragalactic background light (EBL) around us. The EBL is the cumulative sum of all radiation produced over cosmic time, including light from the first stars, galaxies, planets, as well as any truly diffuse extragalactic sources (Fig. 11c) (Hauser and Dwek 2001; Cooray 2016; Tyson 1995). Measurements of the EBL can constrain galaxy formation and the evolution of cosmic structure, provide unique constraints on the epoch of reionization, and allow searches for beyond-standard model physics (Tyson 1995). The absolute brightness of the EBL has been established from Earth at many radio and X-ray wavelengths, but at most infrared (IR), optical, and ultraviolet (UV) wavelengths a precise assessment of the sky brightness has been hampered by reflected and emitted light from IDP, which results in an irreducible $>50\%$ uncertainty (and, at some wavelengths, significantly larger) on the absolute emission from the EBL (e.g., Hauser et al. 1998). At VISIR (0.4–100 μm) wavelengths, the sensitivity of an instrument near Earth is limited by the foreground of scattered light and thermal emission light from the circumsolar dust cloud. Reductions in this bright foreground permit tremendous gains in sensitivity and temporal stability that permit new kinds of observations of both the solar system and the universe beyond it (Zemcov et al. 2018).

## Needed Measurements

### Magnetic Fields

**Fig. 12:**
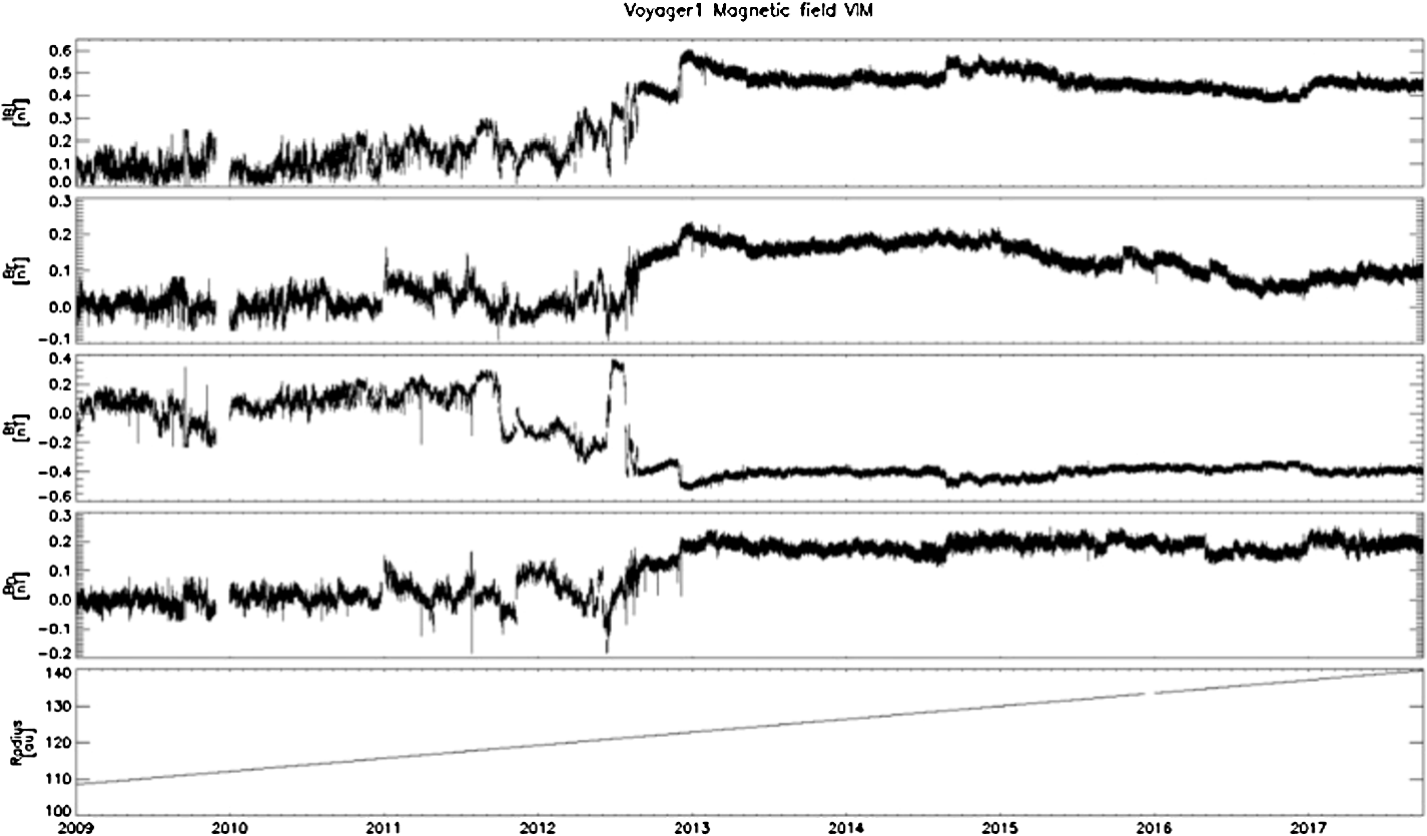
Voyager 1 magnetic field measurements from 2009 to 2018 covering both the inner and outer heliosheath. (Image courtesy of A. Szabo)

The very weak fields in the VLISM are in the sub-nT range with variations in the 1–10 pT range (Fig. 12) and therefore require the use of very offset-stable, low noise magnetometers, within a temperature-stable and magnetically clean environment. Current state-of-the-art fluxgate magnetometers and electronics can achieve noise levels of 2 to 10 pT/sqrt(Hz) at 1 Hz and offset drifts of $<3~\text{nT}$ over a temperature range of more than $100~^{\circ}\text{K}$ and 10 years of operation in space (Bepi Colombo (Heyner et al. 2021), Solar Orbiter (Horbury et al. 2020), MMS (Russell et al. 2016), THEMIS (Auster et al. 2008), and Parker Solar Probe (Bale et al. 2016)). Regular in-flight calibrations are important for achieving offset accuracies of 10’s pT. These can be achieved either by continuous offset calibration making use of Alfvénic magnetic field disturbances (e.g., Belcher 1973; Hedgecock 1975; Leinweber et al. 2008) or compressional fluctuations (e.g., Plaschke and Narita 2016; Plaschke et al. 2017), and/or by routinely performing spacecraft roll maneuvers over two axes (e.g. Dougherty et al. 2004). The inflight calibration routines may be implemented and used on board, to reduce telemetry requirements, after an initial testing and adjusting phase. Further development in this direction would, however, be necessary and feasible, based for instance on the already performed on-board processing of magnetic field measurements on the GEO-KOMPSAT-2A spacecraft (Magnes et al. 2020). Offset calibration accuracies based on a method that makes use of Alfvénic fluctuations have been derived as a function of solar wind measurement time at 1 au (near Earth) by Plaschke (2019): 40 hours of measurements would suffice for accuracies of 0.2 nT at 1 au, but further out the magnetic field is significantly weaker, and the accuracy should scale with it. Within the same amount of time, 20 pT offset accuracy should be achievable in the VLISM if Alfvénic fluctuations are equally prevalent there. Over the calibration interval (e.g., 40 h), the offset drift of the magnetometer/spacecraft system should not exceed the required offset accuracy.

A suitable thermal management concept is required to mitigate temperature-dependent noise levels. Extensive experience in thermal management of magnetometer sensors has been gained from multiple missions to the outer and inner solar system, e.g. JUICE, BepiColombo, Cassini and MESSENGER.

A stringent magnetic cleanliness program has to be implemented for the spacecraft and for all the instruments. Such programs have been successfully executed recently for the Magnetospheric Multiscale (MMS) mission (Russell et al. 2016), for the MESSENGER mission (Anderson et al. 2007) and for the outer heliospheric mission Cassini (Narvaez 2004). In addition, at least two sensors should be included in a gradiometer configuration, with one sensor positioned closer to the spacecraft body (inboard) and another sensor as far away as possible (outboard), at the tip of a long boom. On Voyager 1 and 2, two fluxgate magnetometers mounted on 13-m-long booms were used to observe the differential spacecraft fields; they were sufficient to reach an accuracy of $\sim0.1~\text{nT}$. Occasional spacecraft rolls around the spacecraft–Earth axis provided additional calibration points for two of the three components. The Voyager spacecraft needed to perform such roll maneuvers every 30–60 days to maintain the required accuracy.

Lastly, the required sampling frequency of the magnetometer(s) and joint onboard processing with other measurements (waves and particles) should be discussed. Joint onboard processing would allow for defining and triggering of certain “burst” modes, wherein data would be locally stored at enhanced sampling rates for short amounts of time (e.g. THEMIS, Angelopoulos 2008). This would allow for capturing short but scientifically interesting time intervals in high time resolution without producing prohibitive amounts of data. This approach might reveal aspects of ion kinetics that would otherwise be difficult to detect given the telemetry constraints. Alternatively, burst data could be routinely stored on board and selectively transmitted to Earth based on scientific interest (see scientist in the loop concept at MMS, Burch et al. 2016). For these burst events/data, all involved instruments (also the magnetometers) should be able to measure at significantly higher cadences with respect to the rates that will typically be transmitted to Earth. Sampling rates of 128 Hz are a standard capability of science-grade spacecraft fluxgate magnetometers. A burst mode capability should add but not replace a continuous measurement of data (e.g., at 1 Hz or lower) to be made available to scientists within the constraints of telemetry.

### Radio and Plasma Waves

**Fig. 13:**
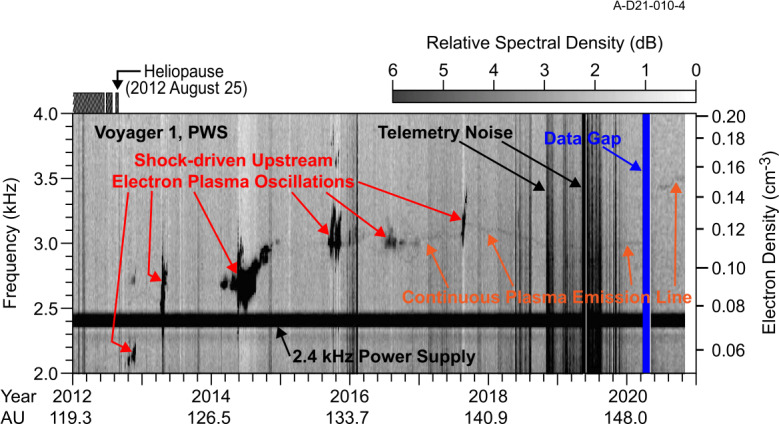
Frequency-time spectrogram from Voyager 1 extending from 2012 into late 2020 showing both intense electron plasma oscillations, highly saturated in this view, and a weak emission at $f_{pe}$. From Gurnett et al. (2021a)

**Fig. 14:**
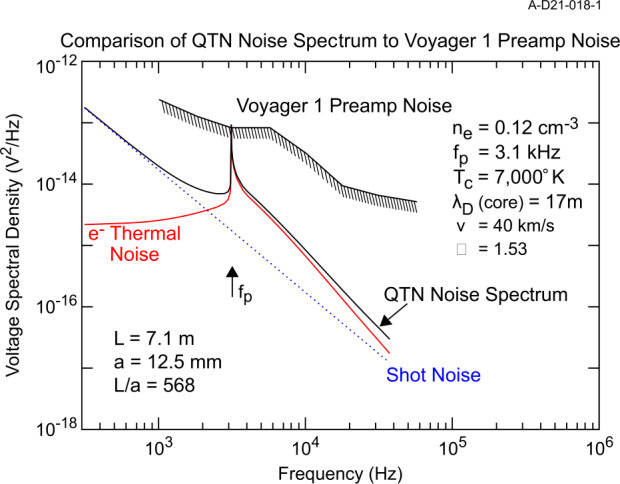
A modeled QTN spectrum using a kappa distribution with $K = 1.53$ showing that such a distribution could produce an emission line at $f_{pe}$ detectable by the Voyager 1 PWS instrument. From Gurnett et al. (2021a)

The only in situ plasma density measurements from the VLISM from Voyager are derived from Plasma Wave Science (PWS) measurements of waves at the electron plasma frequency $f_{pe}~[\text{Hz}] = 8980\sqrt{}n_{e}~[\text{cm}^{-3}]$ (Gurnett et al. 2013; Gurnett and Kurth 2019; Kurth and Gurnett 2020; Gurnett et al. 2021b). There are two types of emissions of interest as illustrated in Fig. 13. The first is an instability at $f_{pe}$ driven by a beam of $\sim100~\text{eV}$ electrons in the electron foreshock of shocks propagating in the VLISM (Gurnett et al. 2021b). These are similar to plasma oscillations observed upstream of planetary bow shocks. While intense and easy to detect, they require the elements of the bump-on-tail instability to be present in order to exist, hence, they are only occasionally observable. On Voyager 1, an event was observed approximately once per year through 2018. A second feature recently observed in the VLISM is a very weak line at $f_{pe}$ (Burlaga et al. 2021; Ocker et al. 2021). Gurnett et al. (2021a) found that this signal is within about 1 dB of the Voyager PWS noise threshold and requires the electric antenna to be nearly aligned with the VLISM magnetic field to be detected by this instrument with only modest length ($\sim7~\text{m}$) antennas. Furthermore, a simple Maxwellian distribution at the VLISM temperature is insufficient to result in a quasi-thermal noise (QTN) signature (cf. Meyer-Vernet and Perche 1989) at $f_{pe}$ that is detectable by the Voyager instrument. Rather, a modeled kappa distribution with $K\sim1.53$ appears to result in a narrow line that is detectable by Voyager as shown in Fig. 14.

The detection of plasma waves at $f_{pe}$ or the quasi-thermal noise signature provides a measure of the electron density that has been used on several missions as the gold-standard by which moments of the electron distribution are calibrated in-flight. Further, a high-resolution QTN spectrum can provide a bi-Maxwellian temperature estimate for the electrons and has even been used to determine bulk velocity. Given that the QTN spectrum requires an effective antenna length that is substantially larger than the Debye length, long antennas would be required for its detection and analysis.

In addition, wave measurements in the few-kHz frequency range provide evidence of the influence of solar transients on the VLISM and, in principle, could reveal evidence of shocks propagating through the ISM from other, non-solar sources. Such measurements were used as early as the early 1980’s to detect radio emissions from solar transients interacting with the VLISM even though the Voyagers were only about 12 au from the Sun and decades from reaching the heliopause.

### Plasma Moments and Composition

Plasma distribution functions and moments (i.e., density, velocity, temperature, pressure) are required for determining fundamental physical processes such as boundary layer physics (e.g., at the TS and HP), plasma wave generation and propagation, wave-particle interactions, plasma turbulence, magnetic reconnection, transients and embedded plasma structures, and many aspects of particle acceleration. Furthermore, resolving composition, such as Li, Be and B might offer a capability for distinguishing solar from interstellar from mixed plasmas in the heliosheath, HP and boundary layer(s), and VLISM. Although challenging due to the low intensities, this could prove to be of high importance for future in-situ observations, particularly considering the extent of the solar system’s influence on the VLISM and fundamental processes such as turbulence, reconnection, and boundary layer physics (e.g., Kelvin–Helmholz instability) along the HP and in the heliosheath, which will be important for determining the requirements for mass resolution for particle instrumentation.

The ability to determine plasma moments for major ions and electrons in the VLISM drives the requirements for plasma measurements. The net ram speed of VLISM plasma (and gas) lies in the range between 30 and 60 km/s depending on spacecraft direction towards the forward hemisphere of the heliosphere (see Sect. 7). This means that the proton energy threshold should start at $\sim5~\text{eV}$ and preferentially even below 3 eV.

The required geometrical factor can be derived from the estimated proton density, temperature, and ram speed in the regions of interest. Assuming a proton density of $0.1~\text{cm}^{3}$, temperature of $8500~^{\circ}\text{K}$, and ram speed of $\sim30~\text{km/s}$ in the VLISM, the differential intensity $js$ would be in the range of $10^{6}~(\text{cm}^{2}\,\text{sr}\,\text{s}\,\text{keV})^{-1}$ at a few eV down to $10^{-2}~(\text{cm}^{2}\,\text{sr}\,\text{s}\,\text{keV})^{-1}$ at a few tens of keV. The expected foreground signal count rate $S$ depends on the energy resolution $dE$ and the geometry factor $Gs: S = js*Gs*dE$. Assuming an energy passband $\Delta\text{E}$ of 10% of the measured energy and a geometry factor of $10^{-3}~\text{cm}^{2}\,\text{sr}$, count rates $S$ of $0.5~\text{s}^{-1}$ and $3\times10^{-5}~\text{s}^{-1}$ can be achieved. With these assumptions, low energies can be resolved with nearly second resolution, while changes in the higher energies will only be notable on the timescale of days. Given that we do not expect fast changes in the VLISM, such long integration may be acceptable.

**Fig. 15:**
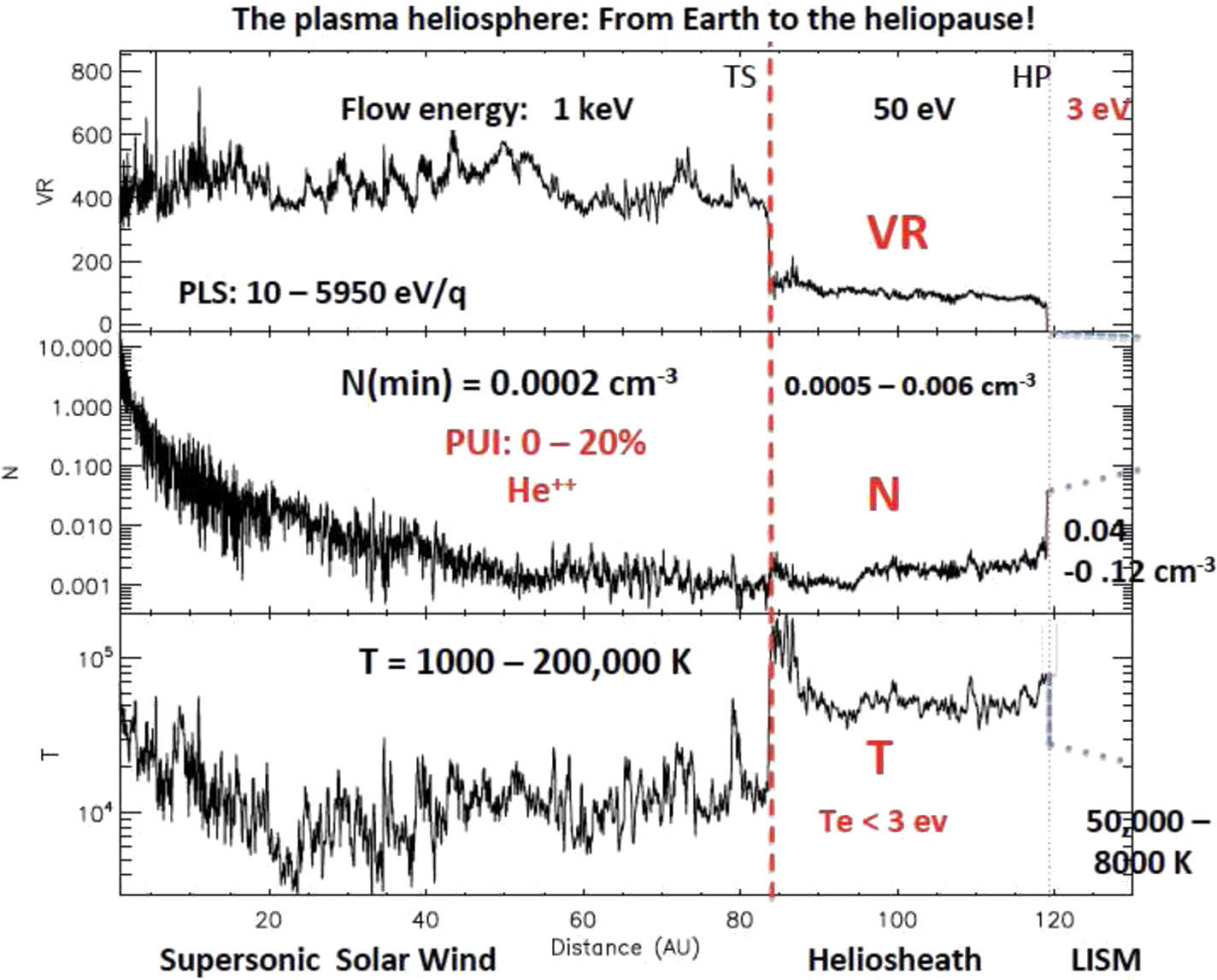
Voyager observations of plasma radial velocity, density, and temperature from Earth to the heliopause. (Figure courtesy of John Richardson, MIT)

Assuming a density of $0.004~\text{cm}^{-3}$, temperature of $10{,}000~^{\circ}\text{K}$, and a ram speed of 100 km/s (Fig. 15; Richardson 2008; Richardson et al. 2019) the intensity at the spectral peak at a few tens of eV will be around $10^{5}~(\text{cm}^{2}\,\text{sr}\,\text{s}\,\text{keV})^{-1}$ equivalent to a count rate of $500~\text{s}^{-1}$ with the assumptions above. With such a rate, one can expect to calculate plasma moments with a time resolution of $\sim1$ second.

Given that flow speeds are so slow in the VLISM, a plasma instrument for Interstellar Probe may also be able to measure PUIs or nonthermal ions in the VLISM. For example, assuming a ^20^Ne density in the VLISM of $3.25\times10^{-5}~\text{cm}^{-3}$ (Table 1 and Gloeckler and Geiss 2004), the ram flux is in the range of $100\text{--}200~\text{cm}^{-2}\,\text{s}^{-1}$ depending on flyout direction. With an estimated temperature of $\sim6300~\text{K}$ and accounting for PUIs occurring up to twice the bulk speed, the resulting differential intensity peaks just below 1 keV would be about $100~(\text{cm}^{2}\,\text{sr}\,\text{s}\,\text{keV})^{-1}$.

**Table 1:** Summary of currently known VLISM parameters at V1, V2 and extrapolations to larger distances

Parameter	Value	Measurement/Model
Interstellar Magnetic Field	$0.48\pm0.04~\text{nT}$	Voyager 1 (Burlaga and Ness 2016)
	$0.68\pm0.03~\text{nT}$	Voyager 2 (Burlaga et al. 2019)
	0.67 nT at HP	IBEX and Cassini (Dialynas et al. 2020)
	$0.293\pm0.008~\text{nT}$ at 1000 au; 227.28^∘^ ± 0.69^∘^ ecliptic longitude, 34.62^∘^ ± 0.45^∘^ ecliptic latitude	(Zirnstein et al. 2016)
	0.37–0.38 nT;	Izmodenov and Alexashov 2020
	(∼204^∘^, ∼43^∘^) ecliptic longitude and latitude at 400–500 au	
Plasma Density	$0.061\pm0.018~\text{cm}^{-3}$ (^1^H)	PUIs (Gloeckler and Geiss 2004)
	$0.0083 \pm 0.0021~\text{cm}^{-3}$ (^4^He)	
	$2.06 \times 10^{-6}~\text{cm}^{-3}$ (^3^He)	
	$4.7 \times 10^{-6}~\text{cm}^{-3}$ (^14^N)	
	$1.5 \times 10^{-5}~\text{cm}^{-3}$ (^16^O)	
	$3.25 \times 10^{-5}~\text{cm}^{-3}$ (^20^Ne)	
	$0.00898 \pm 0.00012~\text{cm}^{-3}$ (He)	(Bzowski et al. 2019)
	$0.12 \pm 0.04~\text{cm}^{-3}$	Hubble Space Telescope (Redfield and Linsky 2008)
	$0.07\pm 0.01~\text{cm}^{-3}$ in LIC	(Slavin and Frisch 2008)
	$0.12~\text{cm}^{-3}$ at 146 au	Voyager 1/PWS (Gurnett and Kurth 2019)
	$0.087~\text{cm}^{-3}\pm 8\%$ at 124.2 au	Voyager 2/PWS (Kurth and Gurnett 2020)
Neutral Density	$0.176 \pm 0.019~\text{cm}^{-3}$ (^1^H)	PUIs (Gloeckler and Geiss 2004)
	$0.0154 \pm 0.0015~\text{cm}^{-3}$ (^4^He)	
	$3.83 \times 10^{-6}~\text{cm}^{-3}$ (^3^He)	
	$1.0 \times 10^{-5}~\text{cm}^{-3}$ (^14^N)	
	$7.6 \times 10^{-6}~\text{cm}^{-3}$ (^16^O)	
	$8.6 \times 10^{-6}~\text{cm}^{-3}$ (^20^Ne)	
	$0.19\text{--}0.20~\text{cm}^{-3}$	(Slavin and Frisch 2008; Frisch et al. 2011)
	$0.2~\text{cm}^{-3}$	(Zank et al. 2013)
	$>0.12~\text{cm}^{-3}$	(Dialynas et al. 2019)
	$0.195 \pm 0.033~\text{cm}^{-3}$	(Swaczyna et al. 2020)
Flow Speed	26.4 km/s	Ulysses (Witte et al. 2004)
	$23.5+ 3.0(-2.0)~\text{km/s}$	IBEX-Lo (Möbius et al. 2012)
	$22.75 \pm 2.8~\text{km/s}$^a^	IBEX-Lo (Bzowski et al. 2012)
	$25.99 + 1.86 (-1.76)~\text{km/s}$	IBEX-Lo (Schwadron et al. 2021)
	$25.86 \pm 0.21~\text{km/s}$^b^	IBEX-Lo (Swaczyna et al. 2022b)
	$23.84 \pm 0.90~\text{km/s}$	HST (Redfield and Linsky 2008)
	23.9 km/s	HST (Linsky et al. 2019)
Flow Direction	(75.4^∘^,−5.2^∘^) ecliptic longitude and latitude	Ulysses (Witte et al. 2004), synopsis (Möbius et al. 2004), (Lallement et al. 2004)
	(79^∘^ ± 3.0^∘^ [−3.5^∘^], −4.98^∘^) ecliptic longitude and latitude	IBEX-Lo (Möbius et al. 2012)
	(79.2^∘^ ± 3.2^∘^,−4.98^∘^) ecliptic longitude and latitude^c^	IBEX-Lo (Bzowski et al. 2012)
	(75.59^∘^ ± 0.23^∘^,−5.14^∘^ ± 0.08^∘^) ecliptic longitude and latitude	(Swaczyna et al. 2022b)
	(75.28^∘^ + 2.27^∘^ [−2.21^∘^], −5.200^∘^ + 0.093^∘^ [−0.085^∘^]) ecliptic longitude and latitude	(Schwadron et al. 2021)
Temperature	$6300^{\circ}\pm340~^{\circ}\text{K}$	Ulysses (Witte et al. 2004)
	$7500^{\circ}\pm 1300~^{\circ}\text{K}$	HST (Redfield and Linsky 2008)
	$6300~^{\circ}\text{K}$	(Bzowski et al. 2012; Möbius et al. 2012)
	$7260^{\circ}\pm270~^{\circ}\text{K}$	Ulysses (Wood et al. 2015)
	7000^∘^–$9500~^{\circ}\text{K}$	IBEX and Ulysses (McComas et al. 2015)
	30,000^∘^–$50{,}000~^{\circ}\text{K}$ VLISM at HP	Voyager 2 (Richardson et al. 2019)
	$T_{ISN} = 7496^{\circ} + 1274^{\circ}$ (−1528^∘^) K	(Schwadron et al. 2021)
	$T_{ISN} = 7450^{\circ}\pm 140~^{\circ}\text{K}$^d^,	(Swaczyna et al. 2022b)
	$T_{VLISM} = 6150~^{\circ}\text{K}$^e^	
Dust Flow Speed	$24\pm12~\text{km/s}$	(Krüger et al. 2015)
Dust Flow Direction	(255^∘^ ± 30^∘^, 13^∘^ ± 4^∘^) ecliptic longitude and latitude	(Strub et al. 2015)
	(289^∘^ + 15^∘^ [−60^∘^], −38^∘^ + 20^∘^ [−25^∘^])^f^ ecliptic longitude and latitude	
Gas-to-Dust Mass Ratio	137–323	(Slavin and Frisch 2008)
	193 + 85 (−57)^g^	(Krüger et al. 2015)

^a^From the acceptable range given in the paper

^b^At the HP, with a slightly increased speed in the pristine VLISM (25.9 km/s) due to elastic collisions

^c^Uncertainty translated from acceptable range of speeds of ISNs

^d^At the HP

^e^Due to removing the heating by elastic collisions given in Swaczyna et al. (2019)

^f^Directional shift in 2005 originally reported by Krüger et al. (2007) and further confirmed by Krüger et al. (2015)

^g^Assuming dust speed of 23.2 km/s

**Table 2:** Summary of outstanding science questions

Outstanding questions	Needed measurements
**How is the heliosphere upheld by the physical processes from the Sun to the VLISM, and how do those globally manifest themselves?**	
How do PUIs evolve and mediate the solar wind from the inner to outer heliosphere?	PUI distributions, thermal to suprathermal solar wind plasma
What are the dominant acceleration and transport processes in the solar wind?	In-situ ion and electron distributions, magnetic field, and turbulent wave spectra
How does neutral interstellar gas interact with the heliosphere?	LOS temperature and velocity of hydrogen in the heliosphereIn-situ neutral gas composition and density
What are the processes and particle origin across the heliosheath that uphold the force balance and their global manifestation?	Particle distribution, composition, and charge states across the heliosheath. ENA imaging from external vantage point Remote radio wave observations Lyman-alpha Doppler imaging
What is the role of reconnection and turbulence in the heliosheath and at the heliopause?	In-situ magnetic and electric fields, thermal plasma and energetic particles
What are the physical processes that control the extent and shape of the ribbon and belt?	ENA imaging from changing vantage point In-situ ion distributions, flows and fields between 90–300 AU
What are the sources and dominant acceleration mechanisms of anomalous cosmic rays (ACRs)?	In-situ suprathermal to ACR spectra, waves and fields across HS
How do PUIs and solar wind plasma interact with the TS?	In-situ thermal to ACR ion distributions and compositionIn-situ thermal to suprathermal electronsMagnetic fields and waves across TS ($\pm2~\text{au}$)
What is the nature and structure of the heliopause?	In situ magnetic fields, plasma to GCRs out to ≥10’s au beyond the HP
**How does the Sun’s activity, the interstellar medium and its possible inhomogeneity influence the dynamics and evolution of the global heliosphere?**	
How is the heliospheric boundary modified by solar dynamics?	ENA imaging, plasma wave observations inside and outside heliosphere Solar wind magnetic fields, plasma, energetic particles
	In-situ magnetic fields, waves, plasma and energetic particles in the heliosheath
What are the extent and impacts of solar disturbances in the VLISM	In-situ magnetic fields, waves, ion and electron measurements from thermal to GCRs
How are GCR intensities modulated by heliospheric shielding, solar cycle, and solar dynamics?	ACR and GCR spectra and composition, and magnetic fields throughout the heliosphere and into VLISM
**How do the current VLISM properties inform our understanding of the evolutionary path of the heliosphere?**	
What is the nature of a bow structure (wave or shock)?	In-situ plasma to non-thermal populations and magnetic field at ion-inertial scales
What are the properties and composition of the “Hydrogen Wall”?	Remote high-resolution Ly-alpha spectra In-situ neutral and plasma properties beyond the HP
Determine the properties, heliospheric filtration, processes and inhomogeneities of the VLISM	In-situ sampling of neutral composition and abundance, interstellar dust, magnetic fields, plasma to non-thermal populations
How does the composition of local interstellar material differ from that of the solar system?	Neutral gas and interstellar dust
Constrain the origin of GCRs and implications on nearby ISM properties	In-situ GCR elemental, isotopic composition and spectra beyond HP

Thermal and suprathermal plasmas (up to tens of kiloelectronvolts) are traditionally measured by Faraday cups (FCs) or electrostatic analyzers (ESAs) to determine the intensity versus energy per unit charge ($E/q$) of incident ions and electrons. When post-acceleration and time-of-flight (TOF) measurements are added after an ESA’s electrostatic deflection to determine an incident ion’s velocity, the ion’s mass, energy, and charge can be uniquely identified. After Voyager 2 crossed the HP, its FCs—the Plasma Subsystem (PLS) (Bridge et al. 1977; Richardson and Wang 2012) were not pointed directly into the ram direction and so the observed currents were close to the instrument threshold, resulting in uncertain estimates of the flow velocity, temperature ($\leq3~\text{eV}$), and density. Maximizing SNR is a high priority for any development of a plasma instrument. A high SNR can be achieved either by a complex ESA that uses several coincidences or by an FC that includes three cups accommodated at appropriate angles.

A future plasma instrument must be sensitive enough to measure the very cold interstellar plasma while still offering the dynamic range required to observe the solar wind. Fortunately, preliminary analysis (see Interstellar Probe Concept Study Report at interstellarprobe.jhuapl.edu) has shown that the expected level of spacecraft charging of a spacecraft in the VLISM is only approximately $+5~\text{V}$ and very steady, meaning that it is feasible to be able to measure the cold interstellar plasma, especially when measuring in the ram direction (accounting for both spacecraft motion and the flow of the interstellar plasma).

### Pick-Up Ions

**Fig. 16:**
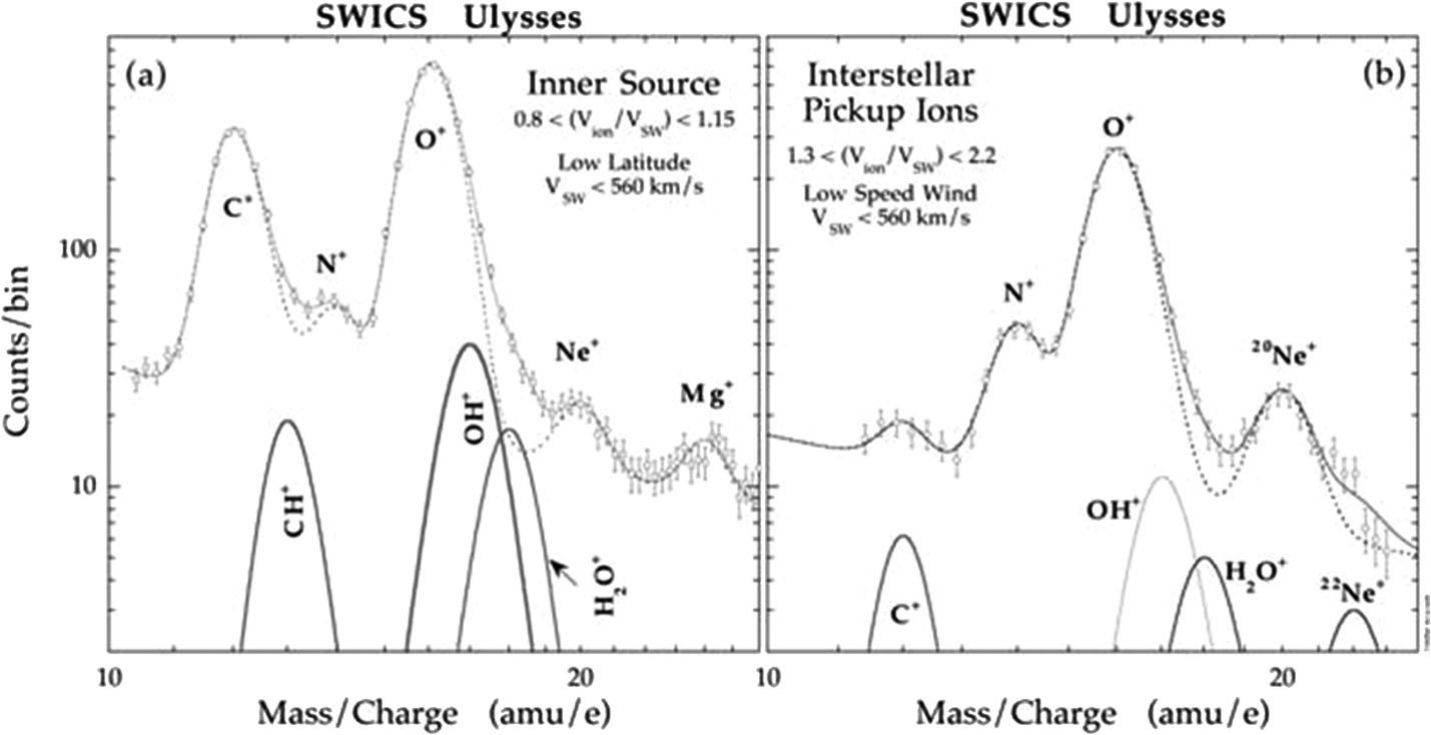
PUI measurements by Ulysses/Solar Wind Ion Composition Spectrometer (SWICS) (reprinted from Geiss and Gloeckler (2001) with permission; © 2001 Springer Nature Limited)

Their crucial role in the dynamics of the outer heliosphere and the VLISM could not be studied with Voyager 1 and 2 because PUIs were and are not measured by those spacecraft. The physics of PUIs within the inner heliosphere has been previously addressed with Ulysses/Solar Wind Ion Composition Spectrometer (SWICS) and Advanced Composition Explorer (ACE)/Solar Wind Ion Composition Spectrometer (SWICS) observations (Allegrini et al. 2005; Geiss et al. 1995; Gloeckler et al. 1992; Gloeckler and Geiss 1996; Schwadron et al. 2000) (Fig. 16), and STEREO PLASTIC observations (Galvin et al. 2008; Drews et al. 2010, 2012, 2015; Möbius et al. 2015; Taut et al. 2018; Bower et al. 2019). While PLASTIC provides 3D velocity distribution function measurements (i.e., arrival directions of ions) for a good fraction of the sunward hemisphere, the full distribution has not been accessible by either instrument, and the small geometric factor of SWICS inhibited progress in understanding the particle processes in the heliosphere. For example, neither the origin nor the production mechanism for “inner-source” PUIs has been established (Allegrini et al. 2005; Gloeckler and Geiss 1996), and although the cosmologically important density of pickup ${}^{3}\text{He}^{+}$ was measured for the first time with Ulysses/SWICS (Gloeckler et al. 1992), this value had a large uncertainty. It is now becoming likely that New Horizons may have sufficient power to be able to observe light PUIs (Kollmann et al. 2019; McComas et al. 2021) (Fig. 16) out through the TS and perhaps some distance into the heliosheath. However, the New Horizons instrumentation was not designed to measure multiple and heavier species of PUIs and the lack of onboard magnetic field measurements will make it difficult to interpret results fully during the crossing of the TS. Any future exploration of the outer heliosphere and VLISM must determine the relative roles between the thermal plasma, PUIs, and the energetic particles in the force balance between the solar wind and plasma in the outer heliosphere and VLISM as well as identify any other thermal populations over the energy range of eV to 100’s of keV, considering also that Voyager left a gap at 5–30 keV.

Protons in the heliosheath with energies in the 1–100 keV range have intensities in the range of 0.1 to $1000~(\text{cm}^{2}\,\text{sr}\,\text{s}\,\text{keV})^{-1}$ (Dialynas et al. 2020) and about a factor of 100 less for $\text{He}^{+}$ PUIs (Krimigis et al. 2003). With a geometry factor of $\sim0.001~\text{cm}^{2}\,\text{sr}$ and 30% energy resolution, we can expect proton count rates between 0.3 and $0.003~\text{s}^{-1}$, meaning that protons can be resolved with a resolution of minutes to hours and $\text{He}^{+}$ PUIs with hours to days.

Proton and $\text{He}^{+}$ intensities in this energy range in the unperturbed VLISM are at least an order of magnitude below heliosheath levels (e.g., Krimigis et al. 2013). Therefore, the challenge quickly becomes to, not only maximize geometry factor, but also SNR. Starting from a functional design such as SWICS (Gloeckler et al. 1998) or similar instruments, and assuming areas for the MCPs and SSDs of $10~\text{cm}^{2}$, a TOF window of 1000 ns, and an SSD pulse rise time of $T = 10~\text{ns}$, one can achieve a triple coincidence rate of $8\times10^{-5}~\text{s}^{-1}$. Based on the count rate estimates above, one can then expect SNR of about 1000 for keV protons and about 10 for keV He ions in the VLISM. To resolve heavier interstellar ions in the VLISM at these low energies, a post-acceleration voltage and a long TOF path are required. A larger geometry factor can be accomplished by an entrance aperture that is larger than the common electrostatic analyzer can afford. Ulysses/SWICS (Gloeckler et al. 1992) implemented such a system, although the geometry factor still made it a challenge to accurately resolve the ^20^Ne and ^22^Ne abundances of interstellar PUIs. A newer iteration of this instrument with current heritage is Solar Orbiter/Heavy Ion Sensor (HIS) (Owen et al. 2020).

An adequate FOV is required to cover both the PUIs within the heliosphere and the net plasma ram once in the VLISM. Although an instantaneous FOV close to $180^{\circ}$ on a spinning platform would cover these directions and provide a full-sky angular coverage, it may be complicated to achieve in the technical implementation. Instead, a two-headed configuration may be preferable to maintain science performance.

Given appropriate geometry factor and SNR, a mass resolution of 20% would allow the Li-Be-B group to be distinguished from He and C. This distinction is critical because it may provide a means to separate solar from VLISM plasma. This is because the abundance of Li-Be-B is five orders of magnitude higher in the VLISM compared to the heliosphere (Wiedenbeck et al. 2007). The ISM abundance is known from cosmic ray abundances in the several to hundreds of MeV range and known sources of Li-Be-B (i.e., cosmic ray spallation in the ISM, as well as dying low-mass stars for Li (Bildsten et al. 1997) (note that fusion in the Sun is *not* a source of Li, Be, or B because of their nuclear binding energies compared to solar temperatures). This mass resolution would also enable other species to be distinguished, such as ^3^He from ^4^He, ^4^He from Li, Li from C, and O from C.

### Energetic Particles

Similar to the LECP experiment (Krimigis et al. 1977) on Voyager, an energetic particle spectrometer would target major energetic ions and their acceleration in the solar wind and heliosheath up through low-energy cosmic rays. Within the heliosheath, energetic ions are an important part of the force balance (Dialynas et al. 2020). The angular and spectral distribution of energetic ions can also be used to derive the bulk flow by applying the Compton–Getting effect (Ipavich 1974) that has been used by LECP to derive flow velocities in the heliosheath (Decker et al. 2012). During the HP encounter, energetic particle measurements will be critical for characterizing the HP and the nature of the instabilities (Krimigis et al. 2019). Beyond the HP, energetic particle measurements will be important for characterizing and discovering any upwind “leakage” into the VLISM (Dialynas et al. 2021) and possibly any energetic ion component in the VLISM.

A lower energy threshold of about 30 keV/nucleon would overlap with plasma and PUI measurements. An upper energy threshold of approximately 10’s MeV would overlap with cosmic ray measurements.

**Fig. 17:**
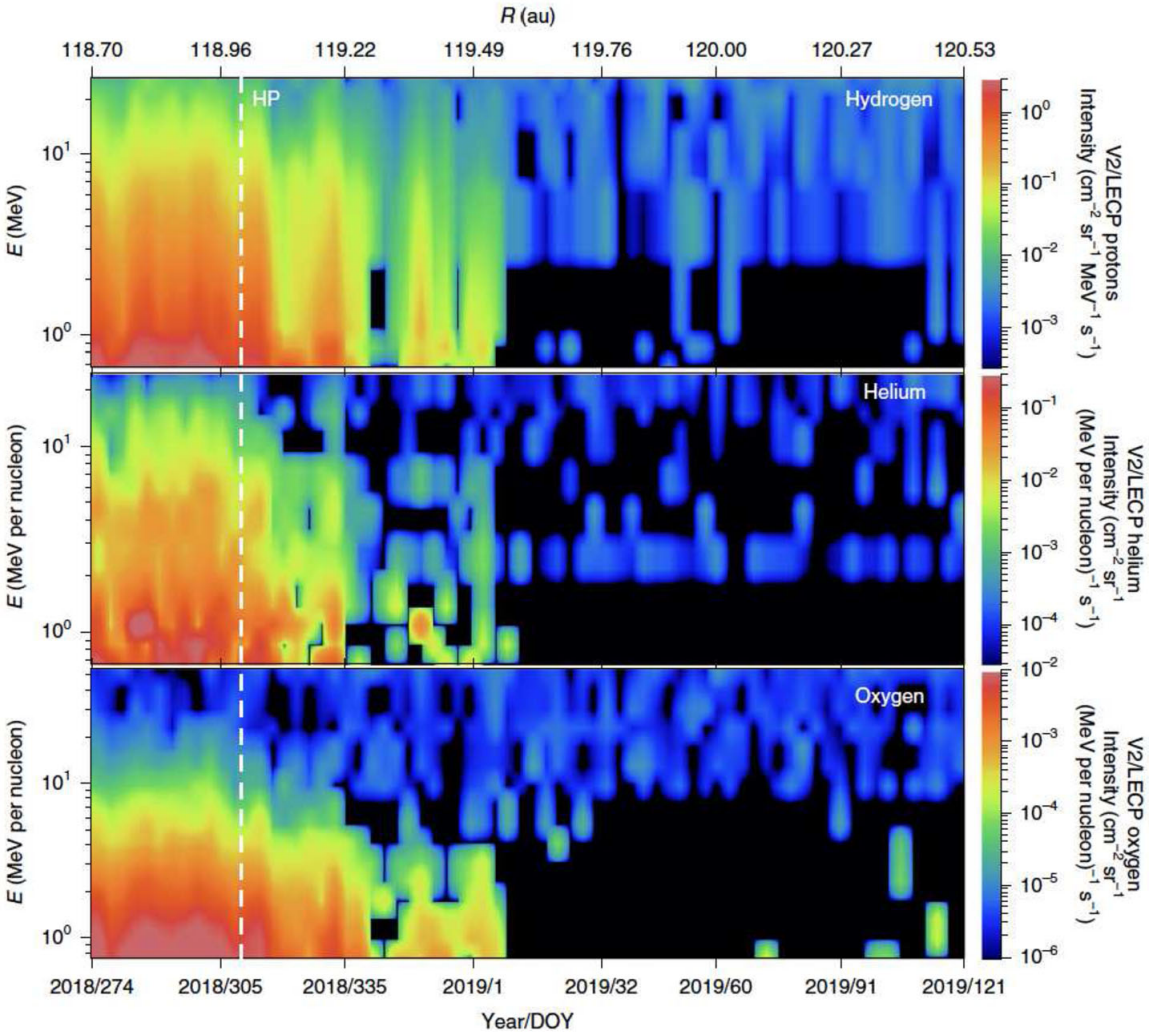
Major ions of the heliosheath as measured by Voyager/Low-Energy Charged Particle (LECP) (reprinted from Krimigis et al. 2019 with permission; © The Author(s), under exclusive licence to Springer Nature Limited)

The primary driver for geometrical factor is to resolve major energetic ions in the VLISM. Just before the HP, the proton intensities detected by LECP on board Voyager 2 were approximately $0.06~(\text{cm}^{2}\,\text{sr}\,\text{s}\,\text{keV})^{-1}$ at $\sim109~\text{keV}$ (Fig. 17; Krimigis et al. 2019; Dialynas et al. 2020), but once in the VLISM, a “leakage” of ions from the HP was detected at about $7\times10^{-4}~(\text{cm}^{2}\,\text{sr}\,\text{s}\,\text{keV})^{-1}$ at $\sim109~\text{keV}$ (Dialynas et al. 2021). Therefore, a total geometrical factor similar to or higher than that of LECP ($\sim0.12~\text{cm}^{2}\,\text{sr}$) is required. It is strongly desired to increase the geometrical factor even up to $\sim1~\text{cm}^{2}\,\text{sr}$ to ensure adequate statistics. Here, one can also make use of the ENA camera (see below), which can successfully be operated as a very sensitive energetic ion spectrometer because of its large geometrical factor.

Energetic electrons up to $\sim1~\text{MeV}$ were measured in the heliosheath by LECP and showed different profiles than those of ions in the heliosheath (Decker et al. 2008). Energetic electron measurements remain a capability that was both achieved by LECP and is achieved by an instrument like EPI-Lo.

At energies above a few tens of keV, a foil-based TOF instrument can be used without the complication of post-acceleration. Mass resolution can be enabled by SSDs so that TOFxE analysis can be performed. Alternatively, a stacked SSD telescope such as LECP (Krimigis et al. 1977) could be used.

TOF-based instruments have been flown or are in development, including EPI-Lo on Parker Solar Probe (Hill et al. 2017), Radiation Belt Storm Probes Ion Composition Experiment (RBSPICE) (Mitchell et al. 2013) on the Van Allen Probes mission, Jupiter Energetic-particle Detector Instrument (JEDI) (Mauk et al. 2017) on the Juno mission, and PEPSSI (McNutt et al. 2008) on the New Horizons mission. The advantage of an EPI-Lo-type instrument is the instantaneous near-hemispheric FOV for maximizing the duty cycle and the fact that it also has a capability to measure energetic electrons. EPI-Lo has been chosen as an example heritage instrument to inform the resource allocations; however, EPI-Lo’s geometrical factor was tailored for the high-intensity region near the Sun and is $\sim0.05~\text{cm}^{2}$ sr summed over all 80 entrance apertures, smaller than the requirement by a factor of two.

### Cosmic Rays

The investigation of cosmic rays starts within the heliosphere with the modulation of ACR and GCR electrons and ions interacting with the solar-cycle variability of the solar wind and solar transient structures, such as coronal mass ejections (CMEs). Combined with PUI and energetic particle observations, cosmic ray measurements target the exact mechanisms of ACR acceleration at the TS and in the heliosheath and test the hypothesis of an offset and asymmetric TS posed by McComas and Schwadron (2006), McComas et al. (2017).

Near the HP, measurements of GCRs and their anisotropies will be crucial for understanding the magnetic barrier and shielding that appear to affect GCRs over distances shorter than their gyroradii (Krimigis et al. 2019). Beyond the HP, observations of the unshielded GCR flux at $<2~\text{GeV/nucleon}$ can be used to determine the sources of GCR acceleration and production by determining elemental and isotopic composition including rare isotopes (e.g., cosmic ray “clocks”) (Ptuskin and Soutoul 1998). Measuring masses in an extended range of GCRs beyond that provided by Voyager to 120 amu (Sn) will provide critical measurements to test and constrain astrophysical models of nuclear synthesis and cosmic ray production. Measurements of anisotropies of GCRs with one or more heads on a spinning platform will provide deeper insight into solar disturbances propagating beyond the HP (e.g., Rankin et al. 2019b; Krimigis et al. 2013, 2019).

Geometry factor is a central requirement for any GCR measurements and is driven by the need to resolve heavier species and rare isotopes beyond the HP in the important energy range of 5 MeV/nuc to a few GeV/nuc. Using the E-$\Delta\text{E}$ technique in a stack of multiple ultrathin silicon detectors, isotopic mass resolution is possible with extremely low background levels. This was demonstrated by Voyager/High-Energy Telescope System (HETS) that provided clean measurements for $<300~\text{MeV/nuc}$ up to 56 nuc with a very low background thanks to the up to eight coincidences applied across its multiple stacked detectors (Stone et al. 1977).

**Fig. 18:**
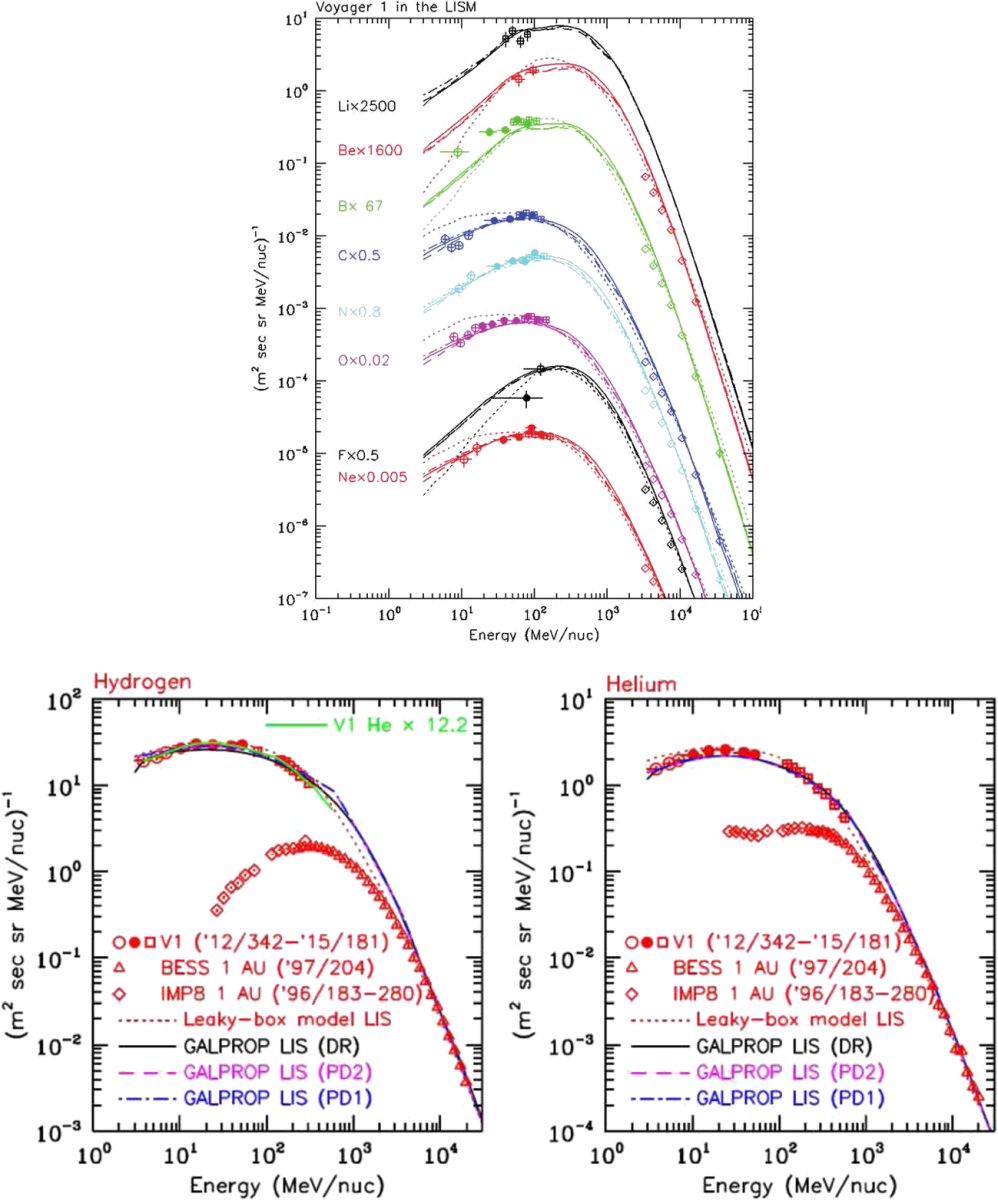
Voyager 1 provided only a few points over a relatively narrow range of energies of the important Li, Be, and B (top panel; reproduced from Cummings et al. 2016 with permission; © AAS). This has left an important gap in the spectrum for constraining the production of light elements in stars and the role of spallation on heavy ions in the VLISM. Note: The intensities of Li, Be, and B are comparable to those of C, N, and O. Cosmic ray proton (hydrogen) and helium spectra in the VLISM from Voyager 1 are shown in the bottom two panels for comparison. (Reproduced from Cummings et al. 2016 with permission; © AAS)

The intensities of elemental species of GCRs in the VLISM range from $\sim10^{-7}$ $(\text{m}^{2}\,\text{s}\,\text{sr}\,\text{MeV/nuc})^{-1}$ for GeV/nuc Ni to $\sim100~(\text{m}^{2}\,\text{s}\,\text{sr}\,\text{MeV/nuc})^{-1}$ for 10-MeV protons, based on the Voyager measurements in the VLISM (Cummings et al. 2016) (Fig. 18). With a nominal geometry factor of $2~\text{cm}^{2}\,\text{sr}$ and an energy resolution of 30%, count rates of 0.1/s to 1/year can be expected, respectively. With a realistic geometry factor of $100\text{--}1000~\text{cm}^{2}\,\text{sr}$, CRS could deliver count rates of $\sim200\text{--}6000$ per minute for protons, 4–100 per minute for helium, and, with longer integration times of up to 400 per day for lithium, $\sim1000$ per day for carbon and $\sim100$ per day for neon. At even longer integration times of 10–100 days (up to nickel) and longer ($\geq1~\text{year}$) for heavier species and rare isotopes, statistically significant counts can still be accumulated with such a large geometrical factor instrument.

Voyager/CRS consists of two nearly perpendicular high-energy telescopes and four low-energy telescopes, all within a mass of 7.5 kg and 5.35 W (Stone et al. 1977). Although Voyager was not a spinning platform, CRS performed excellent measurements of GCR anisotropies. The EPI-Hi instrument (McComas et al. 2016) onboard Parker Solar Probe consists of three solid-state telescopes, each with a different stack of various thickness and diameter detectors to target different species and energy ranges. Ion species are determined very accurately using energy deposited as a function of distance through the detector stacks. While the EPI-Hi design is optimized for solar energetic particles, the concept is applicable to cosmic ray measurements in the outer heliosphere and VLISM. With three large FOVs and a large geometrical factor, the telescopes would cover almost the entire sky over the course of one rotation.

### Interstellar Neutrals

On an outward trajectory through the heliosphere there are unique opportunities to observe the interstellar neutral (ISN) atom composition and velocity distribution using an appropriate neutral mass spectrometer (NMS) and a low-energy ENA camera. Starting already at around 3 au measurements of neutral gas can inform how ISN atoms are ionized and form the so-called ionization cavity around the Sun (Sokół et al. 2019b). All densities of atomic masses from H to Fe should be measured along the trajectory, including the gas temperature for the dominant species H, He, Ne, O, and C. Note that the fractions of neutral C and Fe are predicted to be extremely low in the VLISM (see for example Frisch et al. 2011) with abundances expected to be embedded in dust grains. C and Fe should still be measured to provide at least upper limits on their charge fractions and detect their presence in dust grains whose elemental composition could be measured by an NMS (see Sect. 6.8).

Passing through the outer heliosheath, the so-called secondary neutral distribution of H, He, and O could be sampled in its source region (Kubiak et al. 2016; Park et al. 2016; Bzowski et al. 2017) as its presence weakens and then fades away with distance from the heliopause. Nearing the unperturbed VLISM, the heating and slowing of the primary population by elastic collisions (Swaczyna et al. 2020) would be measured directly for the first time. The access to an increasingly pristine ISN distribution will help untangling the primary and secondary neutral atom distributions and allows one to study the unbiased velocities and temperatures of each species and their composition unaltered by losses on the way into the heliosphere. In addition, any non-thermal features in the ISN distributions, such as the kappa-distribution tails (Livadiotis and McComas 2013; Swaczyna et al. 2019) and temperature anisotropies (Wood et al. 2019) should be detectable, which may have been partially masked or distorted by the simultaneous presence of the secondary neutrals in inner heliosphere observations. In the VLISM beyond the heliopause, the mass spectrometer will measure absolute densities, abundance ratios, and isotopic ratios of all neutral species, as well as ionization states together with an ion sensor. These observations will characterize the chemical and radiation environment in our neighborhood and quantify the filtration of various species in the heliospheric boundary.

**Fig. 19:**
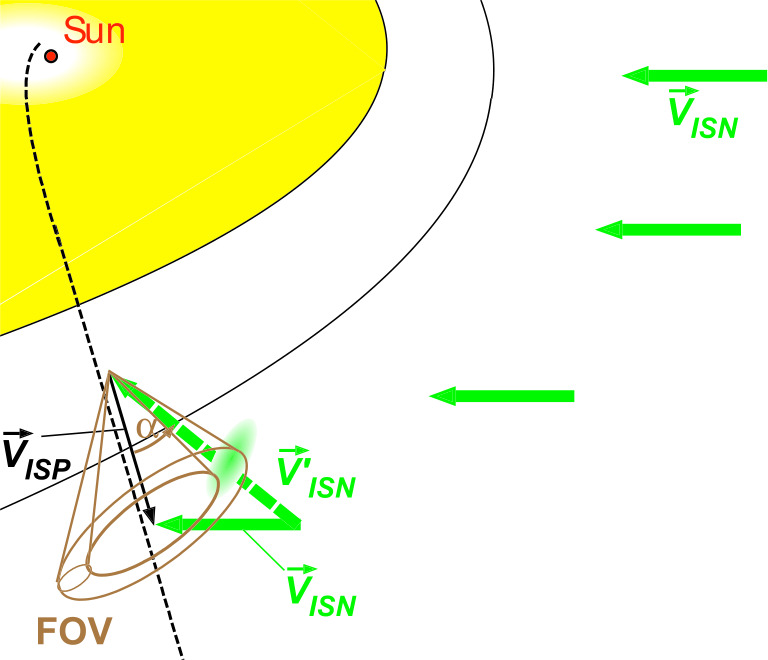
A schematic view of the spacecraft trajectory out of the heliosphere and velocity $\vec{V}_{ISP}$ (black) in the boundary region are shown in the ecliptic plane for mission Option A. $\vec{V}_{ISN}$ (inertial) and $\vec{V'}_{ISN}$ (in S/C frame) are shown in green. The FOV scan of a sensor for the interstellar neutrals is shown in brown pointing with an articulation angle $\alpha $ from the spin axis

To achieve these measurement objectives on a future probe, the neutral gas spectrometer and low-energy ENA camera need to be mounted so that their field-of-view (FOV) scans through the arrival direction of the pristine ISN distribution in the frame of the spacecraft. A sun-pointed spinner greatly facilitates the scan of these single-FOV instruments. Since the energy of the ISN atoms is low, a spacecraft trajectory closer to the upwind direction of the heliosphere is preferred as it results in higher relative speeds of the ISN atoms in the spacecraft frame. Figure 19 shows a schematic view of the arrival direction of the ISN and velocity vector for an example spacecraft trajectory, along with the ISN velocity transformed into the S/C frame and the conical scan of the FOV.

The neutral gas mass spectrometer ionizes incoming neutral particles and accelerates the resulting ions to identify elemental and isotopic abundances of all neutral species in a time-of-flight detector, covering all atomic species up to Fe and even larger molecules or dust fragments. The neutral densities will be measured along the trajectory to obtain timeseries, but only limited information on velocity, temperature, or flow direction of particles will be obtained. The low-energy ENA camera (similar to instruments flown on IBEX (McComas et al. 2009b) and IMAP (McComas et al. 2018)) can measure intensities, energies, and directions of interstellar neutrals and heliosheath ENAs between 10 eV and 1 keV energy, usually distinguishing between the three major species H, He, and O & Ne (the latter two are difficult to separate). In the fixed-angle implementation geometry shown in Fig. 19, these sensors provide conical arcs across the primary and secondary populations of interstellar neutral H, He, and O & Ne, from which spatial and temperature distributions of the different populations can be derived, without the need of a mechanically scanning platform. The NMS alone may provide these for the primary ISN species, but will not be able to observe the secondary neutrals nor any non-thermal features in the distributions. Only an IBEX and IMAP-Lo-like sensor will achieve these objectives.

A low-energy ENA camera will also observe the globally distributed flux (GDF) of ENAs (Schwadron et al. 2011) believed to originate in the inner heliosheath, and the ENA Ribbon (McComas et al. 2009a) that may originate in the region of the outer heliosheath and the near VLISM (Heerikhuisen et al. 2010; McComas et al. 2014; Schwadron and McComas 2013, 2019).

While an actuating platform with a single-FOV low-energy ENA camera would provide a near-hemispheric sky coverage, an accommodation at a fixed angle would provide scans of the ribbon and the GDF radially at an angle along the outward trajectory, complementary to the simultaneous all-sky observations from 1 au with IBEX and IMAP. In the low-energy ENA regime, a direct connection can be established between the original neutral source population, i.e., the GDF that emerge from the inner heliosheath, and the ENA Ribbon at energies below that of the solar wind. This Ribbon source scenario at low energies has provided an independent tool to determine the thickness of the inner heliosheath (Fuselier et al. 2018; Galli et al. 2016, 2017).

### Interstellar and Interplanetary Dust

**Fig. 20:**
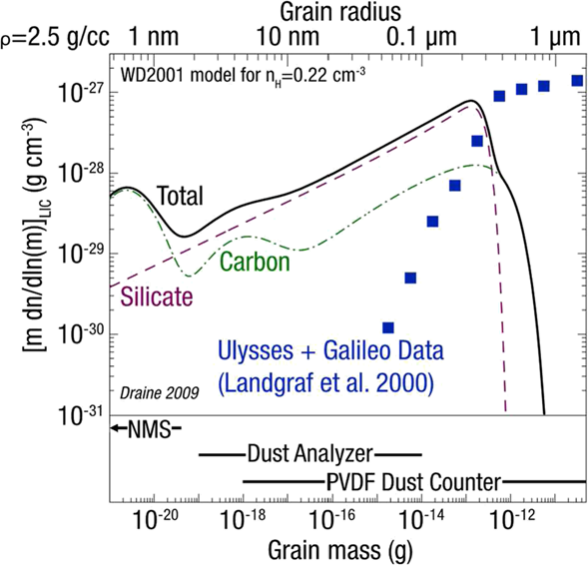
Predicted ISM dust population distribution that Interstellar Probe should encounter (solid black curve from Draine 2009). The dust consists of both silicate and carbonaceous dust grains formed from the thick stellar winds of asymptotic giant branch (AGB) stars and supernovae outflows (Draine 2011). These predictions, based on the best Earth-based remote-sensing measurements of the VLISM, are in strong conflict with the inflowing ISM particles measured in situ by Ulysses and Galileo in the inner heliosphere (Landgraf 2000, blue squares), which resemble much more the 0.3- to 100-μm dust grains found from interplanetary sources. (Adapted from Draine 2009)

ISD grains flow from the local galactic environment through the solar system because of the solar system’s relative motion through the VLISM (e.g., Gruen et al. 1994; Krüger et al. 2007; Krüger and Grün 2009). Understanding the ISD, including its flux, size distribution, directional variability, chemical and isotopic composition remains an unfinished and challenging task (e.g., Krüger et al. 2015; Strub et al. 2015; Sterken et al. 2015) that can be uniquely addressed by a spacecraft traveling through the heliospheric boundaries and into the unperturbed VLISM. ISD grains carry vital information about the original building blocks of our solar system, the electromagnetic structure of the outer heliosphere, the nature and makeup of our local interstellar and galactic environment, and even stellar formation processes (Sterken 2012; Westphal et al. 2014; Altobelli et al. 2016; Horányi et al. 2019; Draine 2009; Landgraf et al. 2002). Because of filtering effects from solar radiation pressure and electromagnetic perturbations from the heliosphere (Slavin et al. 2012; Sterken et al. 2015; Landgraf et al. 2003), the ISD size distribution and composition observed in the inner solar system is highly distorted from that expected in the pristine VLISM and thus remains largely unknown. For example, the size distribution of ISD entering the heliosphere measured by Ulysses (Fig. 20) (Landgraf 2000) contradicts both optical observations and expectations of the required abundance of elements based on current models (Draine 2009, 2011). The bottom panel of Fig. 20 displays the dust grain mass ranges detectable by different instruments, including a dedicated dust analyzer, an NMS, and relatively simple dust counter.

In-situ dust detection would also provide knowledge of interplanetary dust distributions, critical for understanding a multitude of processes throughout the solar system including the formation of the circumsolar dust disk, which, in turn provides insight into the evolution of the solar system. Based on New Horizons measurements, recent modeling suggests that dust generated outside of 30 au from EKB objects and Oort cloud comets accounts for $\sim99\%$ of the total mass of all dust grains in the solar system (Poppe et al. 2019). In other words, the zodiacal light seen from Earth, which is dominated by Jupiter-family comets, comprises only $\sim1\%$ of the picture. Determination of the production rates of IDP grains can inform us about the physical evolution of their parent bodies, including, for example, the fading times of Jupiter-family comets (e.g., Nesvorný et al. 2010) or the current-day collisional state of the Edgeworth-Kuiper Belt (EKB) (e.g., Stern 1995, 1996; Singer et al. 2019). Our solar system’s debris disk also provides “ground-truth” comparison to the multitude of observations of exozodiacal debris disks around other stars (e.g., Trilling et al. 2008; Bryden et al. 2009, 2006; Koerner et al. 2010; Millan-Gabet et al. 2011; Montesinos et al. 2016; Eiroa et al. 2013; Chen et al. 2014; Kral et al. 2017; Hughes et al. 2018; Ertel et al. 2018), where hidden planets may warp and/or perturb their debris disks in manners similar to how Neptune and/or Jupiter may affect the equilibrium distribution of IDP in our solar system (e.g., Liou and Zook 1999; Moro-Martín and Malhotra 2002; Holmes et al. 2003).

**Fig. 21:**
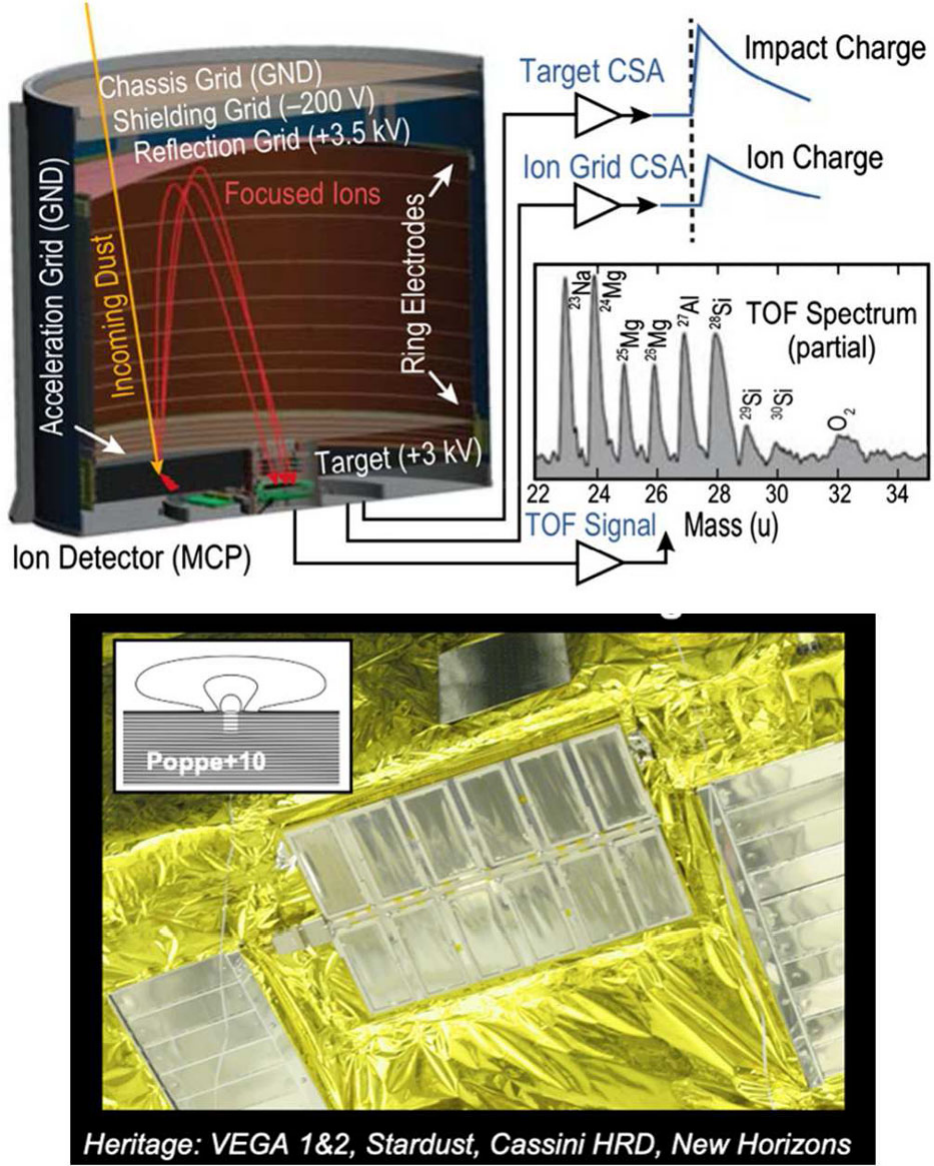
(Left) IDA cutaway diagram of the Interstellar Dust Experiment (IDEX) on the IMAP mission (McComas et al. 2018). IDEX detects dust impacts via impact-ionization-produced charge (left) and concurrently produces high-mass-resolution compositional spectra. (Right) Dust counter for detection of the largest (and thus, rarest) particles impinging on the spacecraft (image credit: NASA, Johns Hopkins Applied Physics Laboratory, Southwest Research Institute). The detector relies on the impact-generated removal of polarized material, creating an electrical signal proportional to the amount of plastic removed (and thus the particle’s mass). A heritage instrument, the Student Dust Counter, has most recently been flown on the New Horizons spacecraft out beyond 50 au (Poppe et al. 2019; Piquette et al. 2019). HRD, High Rate Detector

A dedicated dust composition analyzer relies on time-of-flight (TOF) analysis of the ions resulting from impact ionization of the incoming dust grains on the target surface (Fig. 21) and will therefore measure both the impact rate and the chemical composition of submicron-sized grains. Such an analyzer covers a grain mass range of about $10^{-19}~\text{g}$ to $10^{-14}~\text{g}$, an atomic mass range of 1–500 amu with a resolution of $\text{m/}\Delta\text{m} > 200$ in order to distinguish isotopic variability. Detection of ISDs is maximized when the analyzer points into the velocity vector of ISD flow ($\sim26~\text{km/s}$) as seen from the spacecraft. Because of the low Keplerian speed of IDP grains in the outer solar system (a few km/s), the detection of IDPs is maximized when the analyzer is pointed in the S/C ram direction.

Other instrument types can also detect dust grains and extend the mass range to $10^{-21}$ to $10^{-10}~\text{g}$. The dust counter on board the New Horizons mission (e.g., Piquette et al. 2019) uses thin, lightweight polyvinylidene fluoride (PVDF) films to record dust hits and has provided the most comprehensive dust flux measurements in the outer heliosphere to date. While providing dust flux and basic size distribution, such a detector cannot provide composition measurements, but it can importantly extend the detected range to larger dust particles. Finally, the elemental composition of dust grains with masses less than $10^{-19}~\text{g}$ (“nanodust”) can be determined with an NMS by collecting dust in a foil in front of the aperture that can be heated up to release the dust for subsequent analysis. Lastly, plasma wave antennas are sensitive to the plasma cloud emitted from dust hits on the spacecraft and can therefore record hits of micron-sized dust as was demonstrated on Voyager 1 and 2 (Gurnett et al. 1997).

### ENA Imaging of the Global Heliospheric Interaction

ENAs are produced by charge exchange between singly charged ions and neutral gas, and can be used as a an imaging tool for remotely diagnosing the large-scale morphology and spectra of plasma regions such as planetary magnetospheres (Brandt 2021) and was long hypothesized to also be applicable to the heliospheric boundary (Hsieh et al. 1992; Gruntman et al. 2001). ENA observations of the heliospheric boundary have now been conducted from several missions providing critical information and constraints on the large-scale process and morphology (McComas et al. 2017; Dialynas et al. 2017; Wurz et al. 2008; Brandt et al. 2010).

**Fig. 22:**
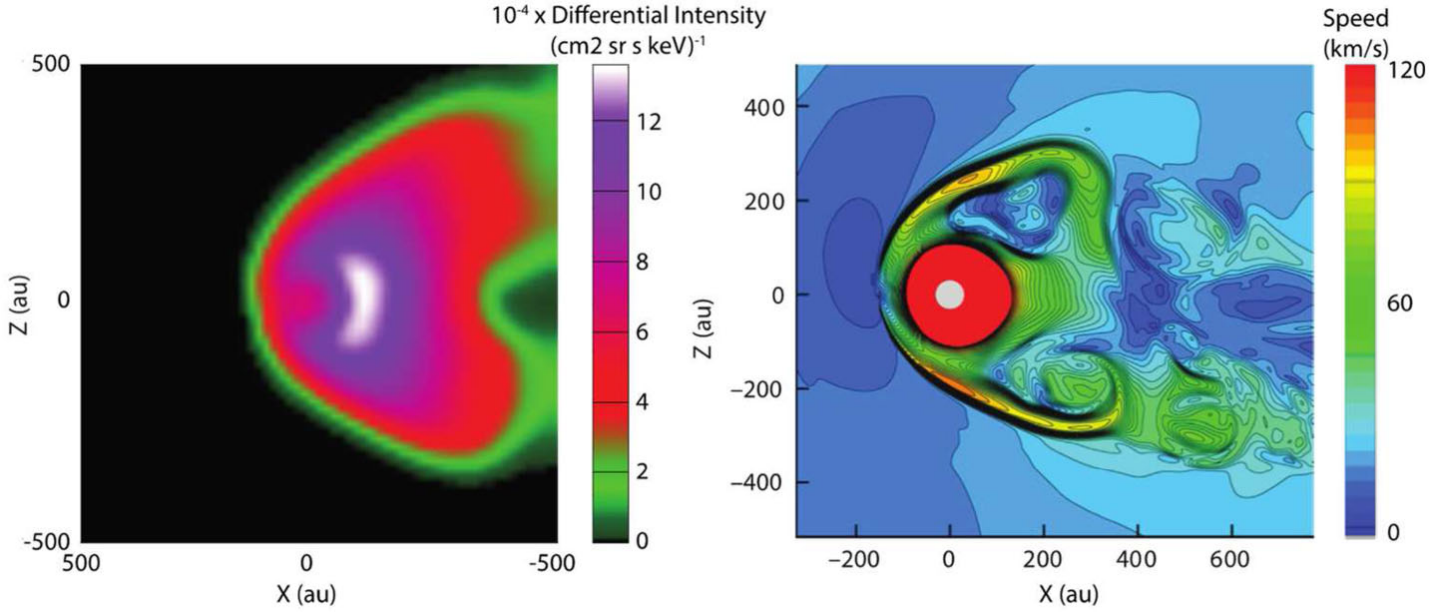
(Left) Simulated H ENA image at 80 keV from a 500 au vantage point $90^{\circ}$ away from the nose direction assuming the flows as modeled by Opher et al. (2020) (right)

In 2025, ENA observations from IMAP (McComas et al. 2018) will take a substantial leap in resolution and imaging capability from its L1 vantage point, which will provide detailed information on the emission features a future mission may fly through. However, the primary objective for a future mission addressing these objectives will be images of the heliosheath acquired from an external vantage point beyond the HP at a distance of about 250 au from the Sun. The expected ENA emissions, other than the ones originating from the ribbon and belt, come from energetic ions (PUIs and non-thermal) convecting through the heliosheath, where ion intensities in this energy range are generally at least an order of magnitude higher (Decker et al. 2005). The ion charge exchange lifetime increases monotonically above 10 keV as the charge-exchange cross-section decreases; thus, the higher-energy ions persist for a much longer time. Therefore, ENA images obtained at lower energies would reveal ions closer to the acceleration source (e.g., the TS), whereas images obtained at higher energies would reveal ions farther away from the acceleration regions, yielding a much better view of the entire shape of the heliosheath. Figure 22 (left) shows a simulated ENA image at 80 keV for hydrogen obtained from a heliocentric distance of 500 au at the flank of the heliosphere with a field of view of $90^{\circ}\times 90^{\circ}$. The distance markers in the simulated ENA image represent the distance to the Sun where the line-of-sight of the simulation intersects the X–Z-plane at $\text{Y}=0$, where the Y-axis is coming out of the page. The simulation uses the velocity field in the heliosheath (Fig. 22, right panel) as modeled by Opher et al. (2020) and assumes the proton spectra above 40 keV directly measured by Voyager 1 (Decker et al. 2005). At energies successively lower than 80 keV, the ENA intensities of the high-latitude jets decrease gradually. At energies closer to solar wind energies ($\sim\text{keV}$), the visible morphology is dominated by the ENA emissions of the neutral solar wind and thus is more spherical (Galli et al. 2019). However, detecting the neutral solar wind would be an important measurement to provide insight into the generation of the ribbon and the generation of PUIs in the VLISM. Therefore, to capture the shape of the heliosphere external imaging should be taken over an energy range from 10 to 100 keV. This results in a very wide dynamic range requirement ($10^{-3}$ to $10^{3}~[\text{cm}^{2}\,\text{s}\,\text{sr}\,\text{keV}]^{-1}$), but can be achieved by long image accumulation times ($\sim\text{weeks}$).

To measure spatial structure on the same scale as the radial thickness of the heliosheath (i.e., $\sim30\text{--}40~\text{au}$), an angular resolution of at least $10^{\circ}$ should be achieved. To view the entire heliosphere, the FOV must be at least $65^{\circ}$ oriented in the anti-ram direction. Characterizing the tail regions on the outward trajectory, however, requires a higher resolution ($5^{\circ}$) because these regions are farther away. Following Schwadron et al. (2014), the structure of the heliotail can be studied at energies between 0.5 and 10 keV with which the approach of “cooling lengths” can be applied. Further, the data presented in Schwadron et al. (2014) show that the tail/lobe regions in the IBEX images extend over most of the downwind hemisphere, suggesting that a very large FOV is desirable. At these energies, solar cycle effects will also play a role in the perceived length of the heliotail (Zirnstein et al. 2020).

From the changing vantage point offered by an outward trajectory, ENA imaging can provide information on the 3D structure of the ribbon observed below about 5 keV (McComas et al. 2009a) and the belt observed above about 5 keV (Krimigis et al. 2009). Assuming an outward spacecraft speed of about 7–8 au/year, single, smear-free images can be accumulated on timescales of a month, which should be sufficient to capture large-scale temporal changes. Measuring the ribbon at $\leq5^{\circ}$ resolution would provide the sampling required to track changes in its apparent width as the vantage point changes along the trajectory. Moreover, depending on the micro-scale processes governing PUI dynamics in the ribbon source region, the 3D structure of the ribbon as viewed by ENA imaging (Zirnstein et al. 2015) may change with the vantage point (Zirnstein et al. 2019).

Using current technology, the energy range could be covered, as it is on the IMAP mission, with a combination of an instrument like IMAP-Hi (0.5–15 keV) and IMAP-Ultra (3–300 keV). IMAP-Hi measures ENAs in a single direction and in a single energy interval at a given time, whereas IMAP-Ultra measures a substantial portion of the sky ($90^{\circ}\times120^{\circ}$) and the entire energy range simultaneously. On the IMAP mission, these cameras both cover the entire sky over large portions of the year through the precession of the spinning spacecraft. However, with a sun-pointed spinner on an outward trajectory, the instruments would not be able take advantage of a change in direction of the spin axis to cover the sky. This is particularly important for an IMAP-Hi-style, single-pixel instrument, which would require a scanning mechanism to cover more than a ring around the spin axis. In contrast, an IMAP-Ultra-like instrument could sweep out a substantial portion of the sky with the fixed spin axis.

### Lyman-alpha Imaging of Interstellar Hydrogen

Measuring solar Lyman-$\alpha $ (1215.67 Å) emission backscattered from interstellar H atoms is a powerful technique to probe interstellar H atoms. A spectral shape of the Lyman-$\alpha $ emission line holds key information on spatial and velocity distribution of interstellar hydrogen that can be used to infer momentum exchange between hydrogen and plasma.

Twenty-five years of Lyman-$\alpha $ observations obtained by Solar Wind Anisotropies (SWAN) onboard the Solar and Heliospheric Observatory (SOHO) from 1 au brought many discoveries: a detection of secondary, warmer, and slower interstellar hydrogen created beyond the heliosphere (Costa et al. 1999); deflection of interstellar hydrogen flow in the heliosphere (Lallement et al. 2005, 2010); variations of the interstellar hydrogen velocity and temperature in the heliosphere due to the solar-cycle effects (Quémerais et al. 2006a); and stability of interstellar hydrogen inflow longitude (Koutroumpa et al. 2017). However, because of the limited spectral data obtained from its hydrogen absorption cell, the SWAN investigation also left many open questions such as: (1) What is a spatial and velocity distribution of the interstellar hydrogen in the heliosphere, and what does it tell us about the charge-exchange coupling at the heliosphere boundary and beyond? (2) What are the effects of various hydrogen populations on the global interaction? (3) What are the structure and properties of the hydrogen wall, and what is the relation to similar structures existing around other astrospheres? (4) Are there any inhomogeneities in the LISM on scales of tens of astronomical units or hundreds of astronomical units?

Measurements on Voyager/Ultraviolet Spectrometer (UVS) (Katushkina et al. 2017) and New Horizons/Alice (Gladstone et al. 2018) showed a surprising behavior of Lyman-$\alpha $ intensity with distance from the Sun, implying a remnant emission of 43 R across the sky that may be attributed to the galactic background (Gladstone et al. 2021).

High-resolution spectra across the sky obtained along an outward trajectory would enable global diagnostics of the non-Maxwellian velocity distribution function of the interstellar hydrogen and therefore understanding of plasma–hydrogen coupling processes in the context of the global heliosphere–LISM interaction. Objectives include (1) determine the properties of interstellar hydrogen flow such as density, velocity, and temperature; (2) discover a position of the hydrogen wall and 3D structure of this unique unexplored global feature; (3) determine the global properties of hot hydrogen atoms created in the heliosheath and their spatial variations; (4) determine a deflection of interstellar hydrogen flow in the heliosphere compared to pristine interstellar flow and discover any deviations from the previously reported deflection of $4^{\circ}$ (Lallement et al. 2005, 2010); and (5) identify galactic and extragalactic components of Lyman-$\alpha $ (Lallement et al. 2011; Gladstone et al. 2018; Katushkina et al. 2017; Gladstone et al. 2021).

**Fig. 23:**
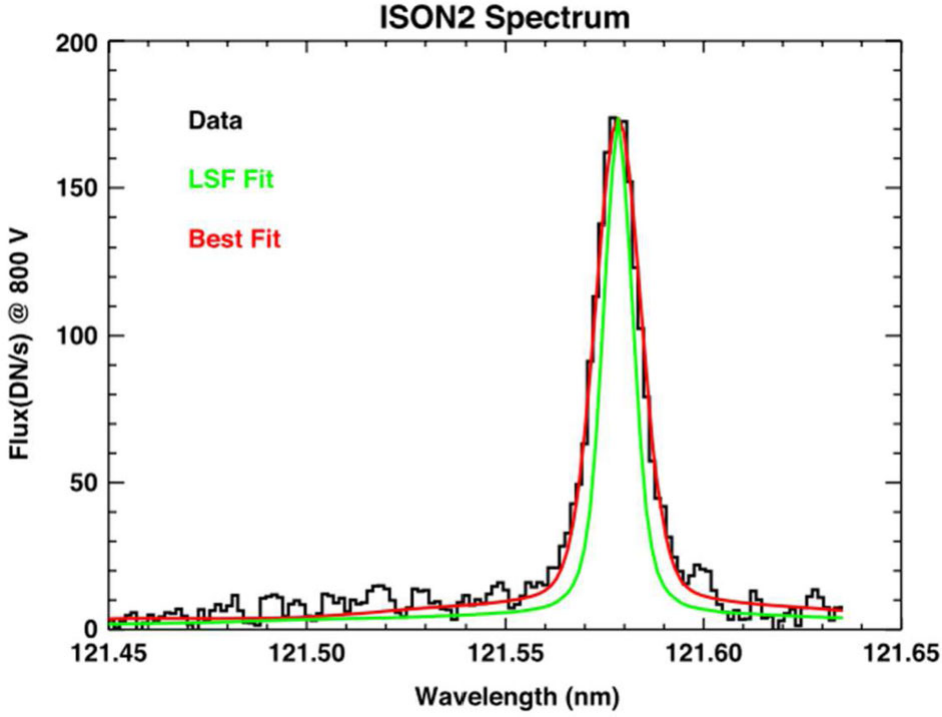
Spectrum of interplanetary hydrogen Lyman-$\alpha $ emission observed by IUVS echelle on MAVEN in December 2013 during the cruise to Mars. The black line is a coadded spectrum from the total 3 hours of integration. Instrument line spread function (green) and best fit to the data (red) are shown. (Figure from Mayyasi et al. 2017)

A Doppler velocity resolution of a few to 10 km/s would be required to resolve the contributions to the Lyman-$\alpha $ emission line from the different hydrogen populations in the heliosheath, hydrogen wall and the pristine VLISM as well as the galactic component. This corresponds to a wavelength resolution of 0.002–0.004 nm or resolving power R ∼ 30,000–60,000. As shown in Fig. 23, a resolution of 0.008 nm was achieved on Mars Atmosphere and Volatile EvolutioN (MAVEN)/Imaging Ultraviolet Spectrograph (IUVS) in echelle mode (Mayyasi et al. 2017). Previous measurements of line widths and line shifts on SOHO/SWAN with the hydrogen absorption cell during half of the solar cycle in 1996–2002 showed noticeable variability of spectral characteristics on yearly timescales (Quémerais et al. 2006a). Therefore, a several-months cadence of spectral measurement will be sufficient to investigate possible variations within a year due to solar effects. To infer spatial variations of line-of-sight hydrogen velocity distributions, multiple look directions are required, in particular toward the nose, toward the tail of the heliosphere, and sidewise, covering at least half of the sky.

Hydrogen absorption cells have been used to reconstruct the Lyman-$\alpha $ line profile from observations on the Prognoz 5 and 6, NOZOMI, and SOHO/SWAN missions (Bertaux et al. 1995). The absorption cell uses its internal H atoms as a narrowband absorption feature. The motion of a spacecraft provides a Doppler shift of this narrow absorption feature against the observed Lyman-$\alpha $ line profile and can be used to scan the line profile by switching the cell on and off (Quémerais et al. 1999, 2000a). While a photodetector approach with a hydrogen absorption cell can be made with low mass, power, and data rates, it only provides indirect information on the line shape and only for a limited range of Doppler shifts controlled by the spacecraft orbit design.

MAVEN/IUVS is an example of a high-resolution spectrograph that includes a far-UV spectral channel that uses an echelle grating to resolve H and D Lyman-$\alpha $ lines. Echelle channels have also been implemented in the spectrographs onboard the Hubble Space Telescope (Clarke et al. 1998). A compact spectrograph design with a high spectral resolution and a high sensitivity is a suitable subject of a future technology development. An alternative to grating spectrographs is an *spatial heterodyne spectrometer* (SHS) with a high resolving power ($\text{R}\sim10^{5}$) and a compact design that recently has been under development for laboratory tests and sounding rocket flights (Harris and Corliss 2018). SHS is a self-scanning Fourier transform spectrometer using a grating serving as a beam-splitter and a dispersing element and mirrors translating beams back to the grating where they interfere and exit the system (Harris et al. 2005).

## A Pragmatic Interstellar Probe for Launch in the 2030s

The idea of an Interstellar Probe to the VLISM is not a new idea and has its roots in a report from 1960 by the Committee of Physics of Fields and Particles in Space to the Space Science Board of the National Academy of Sciences (Simpson et al. 1960). In this report three “Special Probes” were recommended and consisted of a “Solar Probe…to be aimed close to the Sun”, a “Probe ‘perpendicular’ to the ecliptic”, and an “Outer solar system probe: to be aimed away from the Sun in the plane of the ecliptic”. The first two are what later became Parker Solar Probe and Ulysses. Since then, multiple different studies have been conducted including Jaffe et al. 1977, 1980, 1979 suggesting a mission extending 400–1000 au from the Sun toward the nose of the heliosphere with a nominal design life of 20 years and an extended mission to 50 years. Propulsion alternatives included nuclear electric propulsion (NEP) and solar sails to reach the required distances. Initial mass in Earth orbit was estimated to 90,000 kg including the NEP booster with a net spacecraft dry mass of about 1200 kg. The Thousand Astronomical Units (TAU) mission (Nock 1987; Etchegaray 1987) focused on a NEP system powered by a 1 MW nuclear reactor, carrying one astronomy spacecraft and one “spin science and communications” spacecraft, all totaling 5000 kg dry mass.

The Jet Propulsion Laboratory (JPL) in conjunction with a NASA-selected Interstellar Probe Science and Technology Definition Team (STDT) focused on the use of solar sails to achieve a goal of $\sim400~\text{au}$ in 20 years with a requirement of reaching at least 200 au (Liewer et al. 2000). McNutt et al. (2003) outlined a low-cost and lightweight interstellar probe mission and explored the possibility of performing a solar Oberth maneuver (SOM) (Oberth 1929) to reach 1000 au in 50 years.

The “Innovative Interstellar Explorer”, studied under a NASA Vision Mission grant, proposed a $\sim520~\text{kg}$ dry-mass spacecraft with electric power provided by up to six Radioisotope Thermal Generators (RTGs). The study baselined a double gravity assist using conventional chemical propulsion to reach 200 au in 25 years with a projected launch readiness date of 2014 (McNutt et al. 2005; Fiehler and McNutt 2006).

In 2006, a design study of the Interstellar Heliopause (IHP) mission concept (Wimmer-Schweingruber and McNutt 2009a; Wimmer-Schweingruber et al. 2009b) was conducted as a Technology Reference Study (TRS) under the Science Payload & Advanced Concepts Office at ESA. The IHP concept consisted of a 517 kg spacecraft assuming solar sails to reach 200 au in 25 years, and outlined the required development of enabling technologies. Later, in 2014–2015 a series of workshops were conducted Past studies and workshops on the topic *“Science and Enabling Technologies for the Exploration of the Interstellar Medium”* at the Keck Institute for Space Studies (Stone 2015).

The common thread in all of these previous studies has been the compelling core heliophysics science with high-value opportunities for planetary sciences and astrophysics. Their scientific payloads have ranged from sensor suites of 10 kg focused on heliophysics, to payloads of over 300 kg including astronomical telescopes and also separate planetary orbiters. However, the nature of the outstanding scientific questions has remained the same over the past six decades since the Simpson Report.

Most recently, an in-depth study was led by a core team of dozens of scientists and engineers at The Johns Hopkins University Applied Physics Laboratory (APL). The study relied on active involvement from over a hundred international scientists through four dedicated workshops and other meetings. After four years, the study concluded with the Interstellar Probe Mission Concept Report (see interstellarprobe.jhuapl.edu) released in December 2021. Of the past studies referenced above, this NASA-funded study was the most detailed study conducted so far and outlines pragmatic spacecraft designs and mission architectures for a launch in the 2030’s reaching 375 au within a nominal spacecraft lifetime of 50 years with feasible extensions out to 500 au or more. An overview of this study, its trades and conclusions are given below.

### Spacecraft

**Fig. 24:**
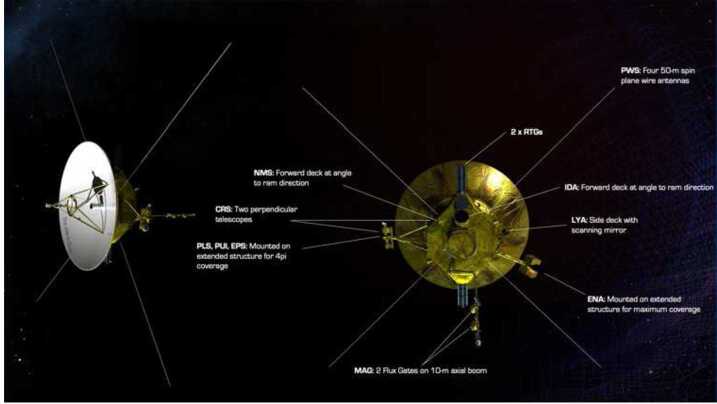
Spacecraft design including the 5-m HGA, two RTGs, magnetometer booms, four spin-plane PWS wire antennas, charged particle suite and ENA camera on pedestals and body mounted instruments

The spacecraft concept (Kinnison et al. 2021) developed for the example payload and target trajectories described below is shown in Fig. 24. The spacecraft bus is a 2-m octagonal structure that supports a 5-m high-gain antenna (HGA), two radioisotope thermoelectric generators (RTGs), and the payload instruments; other spacecraft components are located inside the structure. Interface to the launch vehicle is opposite the HGA. Physically, the system is balanced for spin-stabilized control for the example payload, as the 50-m plasma wave wire antennas require a spinning spacecraft for deployment and control. Instruments are accommodated by mounting them on booms that extend beyond the edge of the HGA to provide clear fields of view. Spinning the spacecraft also allows these fields of view to be swept through $360^{\circ}$ to give the full coverage needed for these measurement types. The spacecraft surface, particularly any obstacles close to the instrument FOVs, needs to be conducting to avoid surface charging that may deflect the measured particles and therefore disturb the measurement.

Electrically, the spacecraft consists of an avionics suite that provides control of all spacecraft systems, interfaces with the payload, and provides for communication with the ground system. As SpaceWire has been adopted throughout the industry, we have chosen to require that all payload and spacecraft components communicate via a redundant, robust SpaceWire bus. Power is generated by two 16-module Next-Generation RTGs (NextGen RTGs) providing in total of 300 W_e_ at the end of the design lifetime of 50 years. The spacecraft system is designed to fit within this anticipated power at end-of-life at 50 years with a 73 W margin assuming all subsystems operating simultaneously.

The principal heat source for the Interstellar Probe spacecraft is the two RTGs. Multi-layer insulation (MLI) covers almost the entire spacecraft, providing a thermos-bottle environment for the internal subsystems, an approach successfully demonstrated on New Horizons. Interstellar Probe will need about 155 W inside the thermal bus to maintain its allowable temperature. Most of the spacecraft components are thermally coupled to the bus. However, instruments mounted off the spacecraft have lower allowable temperature limits, but also draw some heat from the spacecraft and have survival heaters.

The engineering team conducted a significant trade study (Ashtari et al. 2021) to optimize downlink rates, with the goal of providing more than 500 bps at 50 years and downlink rates sufficient to allow significant science at 1000 au. The design for significant downlink rates to 1000 au is not a lifetime requirement on the flight system; rather, it is intended to allow for additional science beyond the 50-year lifetime as flight system performance allows. As a result of this trade, communication is based around an X-band system with multiple antennas, including low-gain antennas (LGAs) used just after launch, a medium-gain antenna (MGA) that can support operations through the inner heliosphere with less stringent pointing requirements, and a large HGA for operations later in the mission at the cost of more restrictive guidance and control requirements to maintain Earth-pointing and optimizing downlink.

The guidance and control (G&C) subsystem maintains the HGA within $0.2^{\circ}$ of Earth and consists of two star trackers that can operate in spin mode, a fully redundant hemispherical gyroscopes, accelerometers, and a sun sensor assembly optimized for spin-mode operations. Actuation is provided solely by attitude control hydrazine thrusters, which are coupled to minimize residual $\Delta\text{V}$. The algorithms and subsystem design are heritage from the New Horizons (Fountain et al. 2008), Van Allen Probes (Stratton et al. 2013), and Interstellar Mapping and Acceleration Probe (IMAP; McComas et al. 2018) missions. Algorithms developed for Van Allen Probes and IMAP will be used to control the system momentum vector that otherwise is problematic due to long flexible booms (Rogers et al. 2021).

### Example Payload

To conduct the needed measurements outlined above and to guide the spacecraft and mission design, a notional, example payload was established within a fixed mass allocation of about 90 kg to reflect successful, historic approaches and also to illustrate the necessary trades that may have to be taken in a future mission. The example heliophysics payload is summarized in Table 3. The resources (mass, power, data rate) required by the instruments were taken from similar flown instrumentation, or in a few cases instruments in development. It has to be noted that individual resource numbers are not allocations, but realistic values of what instruments do require. The scientific balance of the example payload was guided by the dedicated in-situ investigations an Interstellar Probe has to perform, and was complemented by a few powerful remote imaging instruments. An augmented payload was also established to illustrate how the planetary science and astrophysics goals may be met. See the Interstellar Probe Concept Study Report for more details (McNutt et al. 2021).

**Table 3:** Notional, example heliophysics payload used in the study. Total mass and power are 87.4 kg and 86.7 W based on analogous heritage instruments

Instrument	Measurement parameters	Mass	Power	Heritage reference
Magnetometer (MAG)	0.01–100 nT; $\sim10~\text{pT}$ sensitivity; $\leq60~\text{s}$	0.6 kg^a^	5.7 W	MMS/DFG
	For turbulence $10^{-8}~\text{nT}^{2}\text{/Hz}$ sensitivity, 100 Hz sampling	4.2 kg^b^		
Plasma Wave Subsystem (PWS)	$\sim1~\text{Hz}$–5 MHz; Δf/f ≤ 4%; sensitivity ≤0.7 μV/m @ 3 kHz; $\leq60~\text{s}$ ($\leq4~\text{s}$ at TS)	3.3 kg^c^ 8.2 kg^d^	11 W	Van Allen Probes/EFW
Plasma Subsystem (PLS)	$<3~\text{eV/e}$ to 20 keV/e; e, H^+^, He^+^, He^++^, C^+^, N-O^+^; $\leq60~\text{s}$; ∼4*π* coverage	8.0 kg	10 W	PSP/SWEAP or Faraday Cups
Pick-Up Ions (PUI)	0.5–78 keV/e; H, ^2^H, ^3^He, ^4^He, ^6^Li, ^12^C, ^14^N, ^16^O, ^20^Ne, ^22^Ne, Mg, Si, Ar, Fe, charge states; ∼4*π* coverage	5.5 kg	4 W	Ulysses/SWICS, Solar Orbiter/SWA
Energetic Particle Subsystem (EPS)	20 keV–20 MeV; H, ^3^He, ^4^He, Li, C, O, Ne, Mg, Si, Ar, Fe; ∼4*π* coverage; $\leq60~\text{s}$	5.1 kg	5 W	PSP/EPI-Lo
Cosmic Ray Subsystem (CRS)	H to Sn; 10 MeV/nuc–1 GeV/nuc; m/Δm ≥ 10 electrons 1–10 MeV; ≥2 directions; ∼hours	8.0 kg	7.0 kg	PSP/EPI-Hi
Interstellar Dust Analyzer (IDA)	10^−19^ to $10^{-14}~\text{g}$, 1–500 amu; m/Δm ≥ 200, iFOV ≥ 90^∘^	10 kg	12 W	IMAP/IDEX
Neutral Mass Spectrometer (NMS)	H, ^3^He, ^4^He, ^14^N, ^16^O, ^20^Ne, ^22^Ne, ^36^Ar, ^38^Ar, m/Δm ≥ 100; iFOV ≥ 10^∘^; ∼weeks	10 kg	11 W	Luna Resurs/NGMS
Energetic Neutral Atom (ENA) Camera	$\sim1\text{--}100~\text{keV}$ H; iFOV ≥ 170^∘^; ≤5^∘^ resolution	12 kg	9 W	JUICE/JENI, IMAP/Ultra
Ly-alpha Spectrograph (LYA)	$\pm100~\text{km/s}$ Doppler range; $<10~\text{km/s}$ resolution; iFOV ≤ 5^∘^; coverage of upwind and downwind directions; ∼months	12.5 kg	12 W	MAVEN/IUVS
	Other example instrument types part of trade space			
ENA Low-Energy	^e^5 eV – 1 keV; H, D, He, O, Ne; 9^∘^ single-pixel telescope with pivoted FOV	11.5 kg^f^	3.46 W^f^^,^^g^	IBEX-Lo, IMAP-Lo
ENA High-Energy	^e^0.4–15.6 keV; H, He, C/N/O/Ne; 4^∘^ single-pixel telescope	7.37 kg^f^	0.65 W^f^^,^^g^	IBEX-Hi, IMAP-Hi

^a^Sensor and electronics; ^b^Boom; ^c^Sensor and electronics; ^d^Four wire-antennas including deployment mechanisms; ^e^Performance of IMAP-Lo and -Hi; ^f^Resource values from IBEX; ^g^Without central electronics unit

The accommodation of instruments is shown in Fig. 24 and has to meet the general requirements on unobstructed FOVs and look directions for each individual instrument, and meet the requirements on a spin-balanced spacecraft. Two fluxgate magnetometers (MAG) are accommodated on a 10-m boom aligned with the spin axis (the vector normal to and in the center of the HGA), spaced at an appropriate distance apart to capture the magnetic fields accurately and remove any residual spacecraft field. The closest sensor is outside of the spacecraft near-field allowing for the assumption that the spacecraft field is a dipole.

The PWS comprises four 50-m wire boom antennas placed perpendicular to the spacecraft ram direction and $90^{\circ}$ apart from each other, to capture two components of the electric field. These antennas would be deployed shortly after the magnetometer boom, and the spacecraft spin rate ensures the antennas stay properly deployed throughout the remainder of the mission. The antenna deployment would require $\sim1\text{--}2~\text{kg}$ of propellant and is considered a high-risk activity that therefore needs to be performed relatively close to Earth to minimize communication delays. This type of deployment is too risky to be performed beyond tens of au.

The charged particle instruments, PLS, PUI, and EPS, are accommodated out on a rigid pedestal, to maximize the angular coverage and avoid obscuration by the HGA. CRS is accommodated with one telescope on the particle suite boom, pointing $135^{\circ}$ away from spacecraft ram direction, and the other telescope pointing $45^{\circ}$ away from spacecraft ram direction, accommodated on the body of the spacecraft near the base of the particle suite boom. This $90^{\circ}$ angle between the sensors is sufficient to measure the anisotropies expected in cosmic ray detection. The required pointing accuracy for charged particle instruments is assumed to be $\sim1^{\circ}$.

The NMS and IDA are coboresighted and accommodated on the bottom of the spacecraft. Their exact mounting angle relative to the spacecraft ram direction is optimized toward the effective gas and depends on the fly-out direction dust ram flow inflow direction of dust and neutrals assuming a flyout direction. This angle was also chosen to avoid FOV obstructions with other instruments and spacecraft structures such as the magnetometer boom. IDA has a $90^{\circ}$ FOV, and NMS has a $10^{\circ}$ FOV with an antechamber that increases the acceptance cone to $90^{\circ}$. Both instruments are assumed to require a pointing accuracy of $\sim1^{\circ}$.

One ENA camera was accommodated with an energy range of $\sim1\text{--}100~\text{keV}$ and a large instantaneous FOV of about $170^{\circ}\times90^{\circ}$. Other implementations achieve superior performance in the low-energy end of this range, such as IMAP-Lo and -Hi, but use single-pixel telescopes that would require scanning platforms to achieve appropriate angular coverage. The ENA camera was placed on a pedestal to achieve full-sky coverage with a Sun exclusion zone of $20^{\circ}$ cone angle. Two heads may be necessary to achieve this large angular coverage. This pedestal is designed to balance the particle suite pedestal on the opposite side of the spacecraft.

LYA is accommodated next to the ENA pedestal and is placed on the side of the spacecraft pointing away from the Sun. By using an internal mirror scanning $140^{\circ}$ LYA achieves more than a hemispheric sky coverage on the spinning spacecraft and would therefore be able to cover the upwind and downwind directions on any trajectory at an angle away from the nose direction of $40^{\circ}$ or greater. LYA requires a pointing accuracy of $0.4^{\circ}$.

### Mission Design

Three primary methods exist to generate a high-speed departure from the solar system, with each involving some form of a Jupiter gravity assist (JGA). A Ballistic (passive) JGA, using a super heavy-lift four-stage rocket, launching the spacecraft into a direct-to-Jupiter arc with a high-speed transfer (roughly 8–10 months) and perform a low-altitude Jupiter flyby aligned to maximize heliocentric escape speed; a Powered JGA, using a similar super heavy-lift four-stage rocket, deploying three stages during launch to a slightly slower direct-to-Jupiter transfer (roughly 10–14 months) and take the fourth-stage solid rocket motor (SRM) to Jupiter. Then, the SRM fires (creating $\Delta V_{P}$ or a velocity change at perijove) during a low-altitude JGA to enhance the speed gain after the Jupiter flyby; and, lastly a SOM, using a super heavy-lift vehicle, launching the spacecraft with an SRM and a protective solar shield to Jupiter for a retrograde JGA that lowers perihelion to a few solar radii. The SRM executes at perihelion to create orbital conditions with high escape speed. The first option of a ballistic JGA was selected for its competitive solar system exit speed while minimizing spacecraft complexity and mission risk (Schlei et al. 2021).

The intended Interstellar Probe mission trajectory would require a very high C3 range ($200\text{--}400~\text{km}^{2}\text{/s}^{2}$) with either existing or near-existing launch vehicles and upper stages. The Space Launch System (SLS) will provide the highest super heavy-lift performance available with near-existing launch vehicles (Creech et al. 2019; Creech 2019; Stough et al. 2019). Based on experience with New Horizons and Parker Solar Probe, the Atlas V and Delta IV heavy launch vehicles were shown to perform insufficiently to accomplish Interstellar Probe and were therefore eliminated from further study. Several upper-stage configurations with a proven flight record were examined to determine the highest possible launch mass for a likely interstellar probe concept. The highest usable payload system mass is realized by launch vehicle configuration consisting of an SLS Block 2 with an Atlas V Centaur third stage and a Northrop Grumman STAR 48BV fourth stage.

**Fig. 25:**
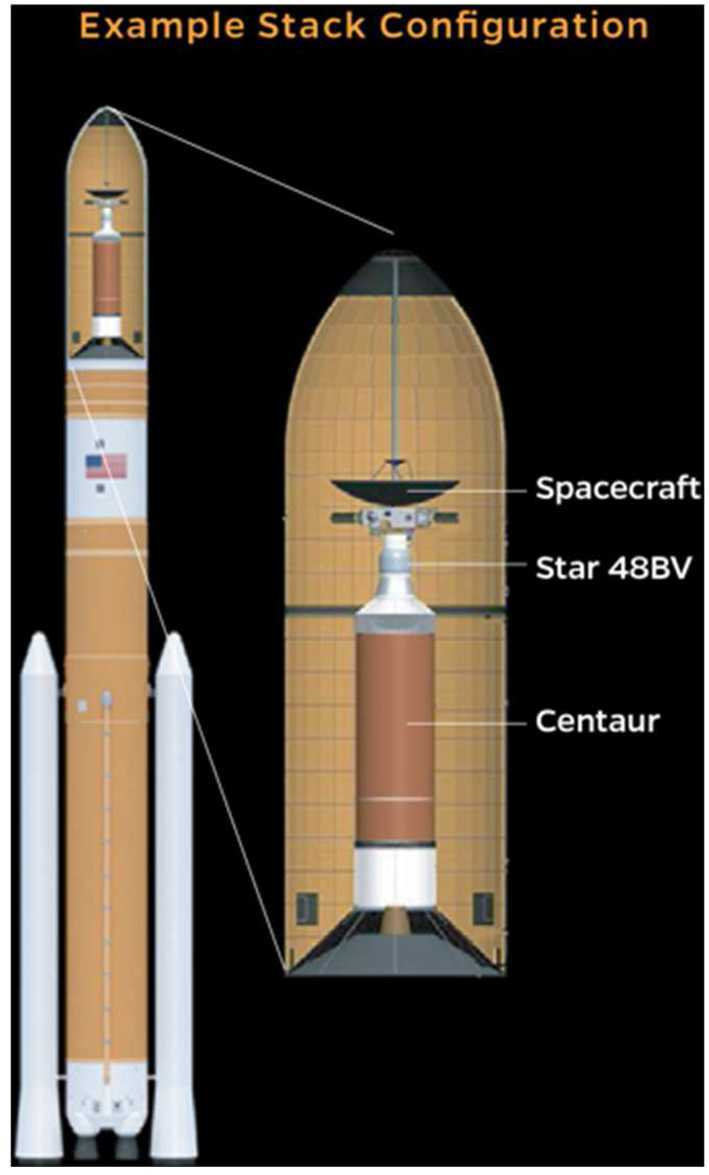
The example stack configuration that maximizes spacecraft mass for a given range of high C3 values consists of an SLS Block 2 with the reference core stage, BOLE boosters, Exploration Upper Stage, Centaur Atlas V and Star-48BV kick stage

Figure 25 shows possible stacks of the stages in an SLS Long Shroud with a significant volume remaining in the long shroud for a spacecraft such as the concept developed for Interstellar Probe.

Other super heavy launch vehicles are in development and may be operational in the time frame needed for Interstellar Probe, including launch vehicles from SpaceX, Blue Origin, and the United Launch Alliance. Because the SLS Block 2 has proven sufficient to meet the requirements of Interstellar Probe and is on track for use before the needed time frame, the SLS has been baselined in this study concept. However, if another launch vehicle does become available and is selected for Interstellar Probe, the mission concept presented here can be performed with no significant modification.

**Fig. 26:**
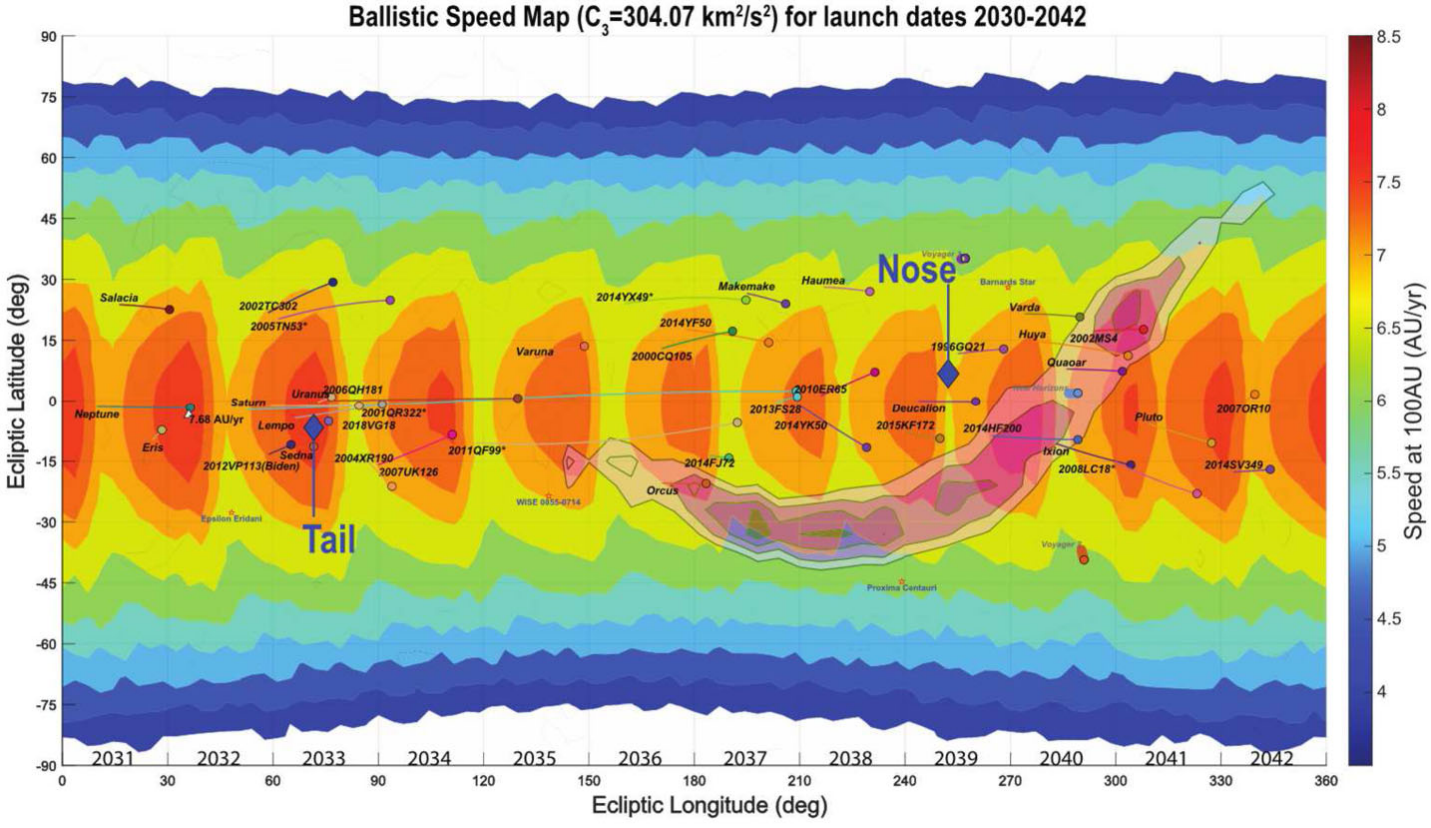
Sky map (ECLIPJ2000) for ballistic JGA cases with $m = 860~\text{kg}$ ($\text{C3} = 304.07~\text{km}^{2}\text{/s}^{2}$) over 2030–2042. The blue to red color-coded contours represent the speed maxima for each year. The purple contours represent the ENA ribbon

Given a likely launch period from 2030 to 2042 (i.e., a complete Jupiter year), a sky map has been generated for each year and aggregated via a maximal speed comparison to produce the trajectory design trade space for a particular option. The sky map in Fig. 26 was constructed assuming a wet mass of 860 kg ($\text{C3} = 304.07~\text{km}^{2}\text{/s}^{2}$) corresponding to the flight system described below. Each possible launch year creates an orange-to-red high-speed zone, or *hot-zone*, to a particular portion of the sky based on the Earth-Jupiter alignment. The purple band depict $45^{\circ}$ off-nose angle with a $\pm5^{\circ}$ width (with the green band showing a $90^{\circ}\pm5^{\circ}$ angle for additional reference). The purple contours represent the ENA intensity values of the 1.1-keV IBEX-Hi channel depicting the ribbon (McComas et al. 2009a).

Four main science drivers dictate the ultimate trade of trajectories:

1. Reach the VLISM within an acceptable mission duration ($<50~\text{years}$)
2. Capture an external side view of the heliosphere to best characterize its shape
3. Travel in the general upwind direction to achieve a net ram speed to facilitate measurements of interstellar gas, dust and plasma
4. Intersect and make in-situ measurement in the ENA ribbon and belt to understand its generation mechanism

Trading these science drivers, and the need to maximize speed, viable launch opportunities begin in 2036 towards the noseward hemisphere of the heliosphere and extend through 2041. For example, a launch in the window of 28 August to 18 September 2036 towards $-22^{\circ}\text{S}$ ecliptic latitude and $180^{\circ}\text{E}$ ecliptic longitude intersects the ribbon and travels at about $80^{\circ}$ off the nose providing an ideal external side view of the heliosphere for ENA imaging (see Fig. 22). The exit speeds within this launch window range from 6.8 au/year to a maximum of 7.0 au/year for a launch on 11 September 2036.

Annual launch opportunities exist throughout the 2036–2041 period that satisfy all of the science drivers with the exception of launch year 2038, when the high-speed region does not intersect with the high intensities of the ribbon. With the general exit speeds around 7.0 au/year over the 2036–2041 period, the net relative ram speeds of interstellar material range from about 30 to 60 km/s and will dictate the accommodation of single-FOV instruments such as a neutral gas spectrometer, dust analyzer and a low-energy ENA detector.

### Concept of Operations

The baseline mission for Interstellar Probe consists of a direct launch to Jupiter with a two-month checkout using the Deep Space Network (DSN) 34-m antennas. During the short cruise to Jupiter the magnetometer boom will be deployed and final instrument commissioning will be completed. For the ballistic option, the 50-m wire plasma wave antennas will also be deployed before JGA. It is estimated that it will take approximately 1 month to deploy the 50-m wire antennas based on the 2-week deployment time for the 50-m wire antennas on the Van Allen Probes and the longer round-trip light time for Interstellar Probe. Instruments will be passively taking science measurements during a ballistic JGA and no specific spacecraft pointing is required. After the Jupiter flyby is complete, the science data will be played back using the DSN 34-m antennas and the spacecraft high-gain antenna (HGA).

The prime science mission starts directly after commissioning and is conducted over the course of three nominal phases, consisting of operation inside the heliosphere ($\sim1\text{--}90~\text{au}$), through the heliosheath/boundary layer(s) ($\sim90\text{--}120~\text{au}$), and into the VLISM itself ($> 120~\text{au}$). The *heliosphere phase* targets observations to answer a range of outstanding questions (Table 1) including the birth and evolution of PUIs, shock acceleration, penetration and interactions with ISNs, heliospheric boundary morphology and dynamics using remote ENA and Ly-a imaging. With a speed of $\sim7.0~\text{au/year}$, the spacecraft will traverse the TS in a little less than 12 years from launch (Table 4).

**Table 4:** Major scientific targets and mission events of an Interstellar Probe and corresponding flight times assuming 7.0 au/year at 100 au

Target/event	Distance	Flight time from launch
Hydrogen ionization cavity	∼3 au	6 months
JGA	∼5 au	9 months
Change in ribbon view	∼10 au	∼1 year
Solar wind slowdown	30 au (5% decrease line) (Elliott et al. 2019)	∼4 years
Termination shock	84 au ± 10 au	11–13 years
Heliopause	120–200 au ± 10 au (Krimigis et al. 2019; Reisenfeld et al. 2021)	17–29 years
Bow wave (existence)/hydrogen wall	200–300 au (Zank et al. 2013)	29–43 years
Unperturbed (pristine) VLISM	∼300–600 au (Izmodenov and Alexashov 2020; Kim et al. 2017)	43–86 years

The *heliosheath phase* is defined to start with the crossing of the TS, where turbulent, small-scale physics may be decisive for the heating of the PUIs. Here, selected burst modes for high-resolution plasma, PUI, and fields and waves measurements may be used and would fit within the available data volume allocation. Selective downlink from high-resolution data stored on the onboard memory can be implemented to maximize the science return. Depending on the heliosheath thickness, the traversal would take 4–9 years and would ensure that large-scale solar-cycle variations can be captured in situ. During this traversal, instrumentation will generally be in the same mode as in the previous phase, with selected and intermittent high-resolution modes during shock encounters and for brief sampling of the turbulent spectra in electric and magnetic fields. Remote ENA observations will continue depending on image patterns (spacecraft may be inside the ENA emitting source region, which may confuse interpretation). Lyman-$\alpha $ observations will continue and will be important for resolving the hydrogen wall from the galactic background. The phase will end with a campaign leading up to the HP crossing and beyond by a few tens of astronomical units.

The *interstellar phase* is chosen to begin once the HP is crossed, as defined by changes in plasma densities, energetic particles, and GCRs, as seen by the Voyagers. All measurements will continue in this phase, including plasma moments, such as flows, densities, and temperatures that will be down to at least 3 eV and perhaps lower. The lower energy threshold of direct plasma measurements will be limited by the spacecraft potential, but beyond the HP, the spacecraft potential may be as low as $+5~\text{V}$ because of the ion deposition being higher than the electron deposition. The positive potential implies that one would be able to measure the plasma electron distribution and estimate the electron temperature. By using analysis of the QTN obtained by the plasma wave antennas, one would obtain an independent estimate of electron density and temperature as well. Intermittent, brief intervals of high-resolution magnetic field and wave measurements can be made to sample the turbulence spectrum. Remote ENA and Lyman-$\alpha $ imaging would continue and would be particularly important given the vantage point far away from the Sun that would provide the first external view of our heliosphere in ENAs, and Lyman-$\alpha $ observations would continue to investigate signatures of the hydrogen wall and possible features from interstellar clouds. The first direct sampling of the unperturbed interstellar plasma, neutral gas, dust, and GCRs will be particularly important in this phase to understand our local neighborhood.

No one really knows how far the heliosphere extends in all directions, and the completely unperturbed VLISM may start already at $\sim300~\text{au}$ or extend to 600 au (Izmodenov and Alexashov 2020; Kim et al. 2017). Within the design life of 50 years, Interstellar Probe would reach about 375 au, more than twice the projected distance of Voyager 1. Operations can be expected out to about 550 au with the current implementation of two RTGs. Beyond this distance, the possibility of operations will become limited by the RTG lifetime. The communications subsystems have been sized for operations at 1000 au to provide data rates meaningful for science.

Science data will be downlinked using three 8-hour contacts per week using DSN 34-m antennas until Interstellar Probe reaches 70 au. At this point communication will switch to using the Next Generation Very Large Array (ngVLA), which will enable a data volume 1.8 Gbit/week at 70 au assuming 8-hour contacts every 2 weeks. The downlink data volume reduces from 1.1 Gbit/week at the beginning of the heliosheath phase to 0.6 Gbit/week at 120 au. After 50 years, Interstellar Probe will still be downlinking 0.142 Gbit/week using 8-hour contacts once per week.

**Fig. 27:**
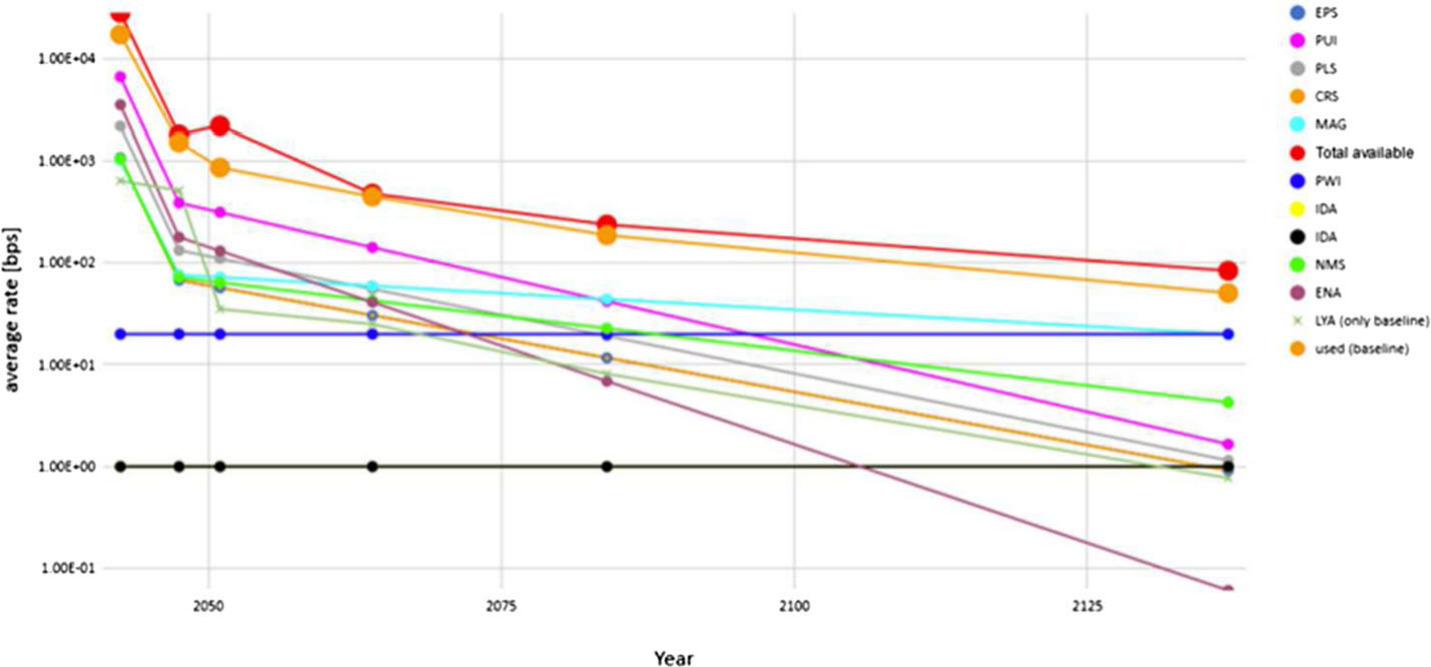
Data rates for each instrument as a function of time. These rates are not allocations. Rates in the early mission are based on what has been used within the solar system, while rates in and beyond the outer heliosphere are representative of what is required to address the science. Summing up these rates (orange) demonstrates that the available downlink capacity (red) is sufficient to perform the required science. Note the increase in available downlink capacity around 2050 is concurrent with the switch to the Next Generation Very Large Array (ngVLA). (Image credit: Johns Hopkins Applied Physics Laboratory)

The available downlink data rate is represented by the red curve in Fig. 27. The data rates of each instrument were bounded using realistic values for the beginning and end of the mission and are meant to demonstrate feasibility and are not allocations at this point. Instrument data rates are based on what has been used when operating similar instruments within the solar system. To bound the representative rates, a useful range for each instrument is determined using the rate needed for Voyager-equivalent science at the low end and a nominal operating rate based on heritage instruments making comparable measurements near the beginning of the mission at the high end. The total data rates of all instruments (orange curve) are within what is available (red curve), even though instruments will need to run at or close to the minimum established for the respective regions. Overall, the available data rates are sufficient to achieve the contemplated science goals throughout the entire mission.

### Longevity

Reaching the local interstellar medium to complete the science objectives for a mission like Interstellar Probe is going to require a spacecraft, a ground system, and an organizational structure that can not only last, but perform well for at least 50 years. These topics collectively are referred to as “longevity” in the context of the Interstellar Probe study.

A statistical analysis (Edwards et al. 2021) identifying 179 spacecraft was conducted to make inferences about a mission that must last at least 50 years. Excluding launch vehicle failures, the historical probability for mission success is 0.94. The most common reasons for the end of long missions include depletion of fuel and intentional termination. With regards to non-intentional failures, but include failures related to the propulsion; telemetry, tracking, and command (TTC); battery; and guidance and control (G&C) subsystems. Overall, technical failures are often not the reason for a spacecraft’s retirement; rather, many spacecraft operate nominally until either a resource is depleted or institutional support for the mission wanes. This suggests that historic spacecraft operational lifetimes frequently reflect intentional mission design decisions rather than poor reliability or limits in engineering capability.

The analysis of design life versus actual life analysis depicts a group of spacecraft that have outlived their design lives by an average factor of seven, illustrating that design life is not an indicator of actual lifetime. Interestingly, major limiting factors to design life include mass and cost, even though much longer actual lifetimes are technologically achievable (Saleh et al. 2006).

To calculate a mean time to failure (MTTF) a reliability analysis was conducted using input data from historic spacecraft performance and expected lifetimes of multiple electronic components. More specifically, a maximum likelihood estimation method was used to fit a Weibull distribution to the data. Using this Weibull distribution, the MTTF was calculated to be 53 years, strongly supporting a choice a 50-year design life of Interstellar Probe.

The probability of mission success was determined as part of the overall mission reliability analysis and to inform choices of instrument redundancy. To do this a Boolean fault-tree analysis was performed with a detailed Science Traceability Matrix (STM). NASA defines several criteria to evaluate missions: baseline mission requirements, threshold mission requirements, and mission success criteria. Baseline mission requirements are simply the set of objectives that the mission is designed to accomplish, and they are established early in the design cycle. The threshold mission requirements are the minimum set of objectives for a mission that must be accomplished for the mission to be worth launching. Mission success criteria are different in that this is the set of objectives that must be completed for the mission to be declared a success, a determination that is made after launch, usually at the end of a mission. Mission success criteria was defined as meeting minimum number of objectives in addressing a particular science question. This logical approach means there are many combinations of objectives leading to mission success, which is appropriate for a mission with such a vast range of exploration and science questions. When analyzing the objective fault tree, over 400 combinations of instrument failures leading to loss of a science question were identified, from which redundancy requirements could be determined. For example, the magnetometer was an instrument that, if lost would fail the mission and therefore should be designed to be redundant. With this approach a notional payload with more than 90% reliability could be constructed and including also the spacecraft, an overall mission reliability of 74% could be established. This process has been used to great effect with the Parker Solar Probe mission (Smith and Kinnison 2021).

## Summary

The outer heliosphere and VLISM represent an almost unexplored region of space and a new realm that heliophysics inevitably is expanding into. Their future exploration will not only open the window to a new regime of space physics, but also provide insights into the past and future of our habitable heliosphere and how its path through the galaxy has helped shape the solar system. Unique interactions between the heliosphere and the VLISM dictate the penetration of interstellar GCRs, gas and dust, and how the heliosphere has globally responded to the dramatically different interstellar environments in the past. Evidence shows that very dense interstellar clouds have encountered the heliosphere and that supernovae have occurred relatively close to the Sun, resulting in the full exposure of the inner solar system to the interstellar environment that may have had far-reaching implications for atmospheric chemistry and perhaps even biological evolution.

The physical processes responsible for upholding the heliosphere and the nature of the VLISM remain the most outstanding problem of space physics today. The little that is known about the heliospheric interaction has been obtained by the Voyager 1 and 2 spacecraft. Because of their limited payload, most notably the lack of PUI measurements, dedicated plasma, dust and neutral gas measurements, many of the discoveries that have resulted have also uncovered mysteries yet to be solved. New Horizons measures proton and $\text{He}^{+}$ PUIs in the outer heliosphere, and is projected to last through the crossing of the TS and only a limited distance into the heliosheath until its power falls below critical levels. Remote information on the heliospheric boundary and VLISM has also been collected by several missions deep in the heliosphere.

A NASA-funded four-year study was recently concluded demonstrating how the groundbreaking science can be achieved from a future, pragmatic Interstellar Probe mission. With an example payload around 90 kg, the mission would explore how the expanding solar wind and its interaction with the VLISM upholds the entire heliosphere, and venture out into the VLISM to directly sample its properties for the first time. Based on current performance of spacecraft subsystems, a nominal design lifetime of 50 years can be achieved, and would deliver an Interstellar Probe out to about 375 au using conventional propulsion and super-heavy lift launch vehicles, such as the SLS Block 2, or other upcoming vehicles from other providers. Launch windows open in 2036 to the forward hemisphere of the heliosphere spanning through 2041. With current technology and RTG power supplies, such a probe would last out to 550 au, more than three times farther than any spacecraft has gone before. Progress in RTG technology remains the main limitation on achieving an operational life out to 1000 au.

The final study report has been submitted to the Solar and Space Physics Decadal, which is in session 2022 through 2023. The community-driven committee and panels are expected to provide final recommendations on NASA’s next heliophysics missions in late 2023 or early 2024. Interstellar Probe would not only enable discoveries in a new regime of space physics for understanding our habitable astrosphere, but would also represent the expansion of space physics and robotic space exploration.
